# Programmable 3D Photovoltaics via Mechanically Origami‐Coded Interlocked 3D Kirigami and Nano‐Root Anchored AgNWs–In–Ga Multiphasic Alloy Conductor

**DOI:** 10.1002/adma.202523685

**Published:** 2026-06-09

**Authors:** Seok Joon Hwang, Jiwon Ryu, Byungsoo Kang, Injong Oh, Dae‐Hee Cho, YoungHoi Cho, Seung S. Lee, Dong Hoe Kim, Deokjae Choi, Gee Yeong Kim, Heesuk Jung, Taehee Kim, Hyeonggeun Yu, Seungjun Chung, Byoung Koun Min, Phillip Lee

**Affiliations:** ^1^ Sustainable Energy Research Division Korea Institute of Science and Technology (KIST) Seoul Republic of Korea; ^2^ Department of Materials Science and Engineering Korea University Seoul Republic of Korea; ^3^ Department of Mechanical Engineering Korea Advanced Institute of Science and Technology (KAIST) Daejeon Republic of Korea; ^4^ Department of Chemistry Sungkyunkwan University Suwon Republic of Korea; ^5^ KIST‐SKKU Carbon‐Neutral Research Center SKKU Suwon Republic of Korea; ^6^ Division of Nanoscience and Technology University of Science and Technology (UST), KIST School Seoul Republic of Korea; ^7^ School of Electrical Engineering Korea University Seoul Republic of Korea

**Keywords:** nano‐root anchored AgNWs‐In‐Ga multiphasic alloy, programmable 3D kirigami, self‐folding solar modules, ultrahigh effective areal coverage photovoltaics, ultrastretchable energy systems

## Abstract

3D photovoltaics (3DPVs) are highly promising for next‐generation energy systems, as they maximize space utilization and power output. However, most reported 3DPVs remain unsuitable for deformable applications. When 3DPVs are integrated with mechanically deformable platforms, a fundamental trade‐off emerges: achieving large mechanical stretchability typically leads to a reduction in areal coverage. Here, we present a single‐material, mechanically origami‐coded 3D kirigami platform that enables programmable 3DPVs with unprecedented stretchability and areal coverage. Origami, which transforms 2D geometries into 3D forms, provides an effective structural strategy to overcome the long‐standing trade‐off between stretchability and areal coverage. Leveraging this concept, we introduce an origami‐inspired mechanical coding scheme that embeds programmable folding behavior into the structure. A simple extension–release cycle drives unit folding initiation and full origami activation, achieving ultrahigh stretchability (500%) and initial effective areal coverage (225%), while maintaining stable photovoltaic output under extreme deformation and repeated cycling. To ensure reliable electrical integration, an intrinsically integrable nano‐root anchored AgNWs–In–Ga multiphasic alloy conductor was co‐fabricated with the structure, providing stable conductivity and enabling reversible stack‐and‐connect operation. This approach highlights geometric programmability as a key enabler for freeform photovoltaics, establishing a pathway toward multifunctional 3D energy systems for adaptive devices, and urban energy harvesting.

## Graphical abstract

Mechanically origami‐coded 3D kirigami photovoltaics convert a single extension–release cycle into deterministic self‐folding, reconfiguring planar solar cells into programmable 3D modules. Distinct coding strategies enable either ultrahigh stretchability (500% system strain) or ultrahigh initial effective areal coverage (225%). A nano‐root anchored AgNWs–In–Ga multiphasic alloy electrode preserves electrical continuity during extreme deformation and repeated cycling.

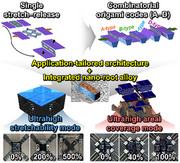

## 1 Introduction

Maximizing device performance and overcoming existing limitations have increasingly highlighted 3D structuring as a critical strategy. By enhancing spatial utilization and maximizing integration density within a confined footprint, 3D architecture has demonstrated significant performance improvements across diverse applications, including electronic devices, energy systems, sensors, and communication platforms [1, 2, 3].

In photovoltaics (PVs), where output characteristics generally increase with the exposed illuminated area, simply arranging solar cells into 3D configurations can increase the effective active area and projected power output per unit footprint under specific geometries and illumination conditions. Indeed, 3D photovoltaics (3DPVs), reconstructed from conventional 2D solar cells, have been reported to enhance power output per unit footprint relative to planar counterparts in certain experimental configurations [4, 5, 6, 7, 8, 9]. However, most reported 3DPVs remain confined to pre‐folded or static configurations, missing structural strategies for stretchability and adaptability to diverse forms and conditions, as required in distributed PV systems and wearable PVs.

Attempts to directly manufacture energy devices in 3D have been explored [10], yet such approaches are often constrained by fabrication complexity, limited compatibility with established device processes, and challenges in integrating reliable electrical interconnects within 3D architectures. Moreover, PV technologies are inherently optimized for 2D deposition and printing processes, making them fundamentally planar. Consequently, rather than pursuing new 3D manufacturing, a more practical and effective strategy lies in reconfiguring mature 2D PV technologies into 3D arrangements.

Origami provides a highly efficient approach for this transformation, enabling precise and reversible conversion of 2D PV films into functional 3D architectures [11]. Beyond geometric transformation, origami serves as an effective structural strategy for imparting mechanical compliance and reconfigurability, enabling energy platforms that overcome the static nature of conventional 3DPVs. However, conventional self‐folding strategies often rely on shape‐memory polymers [12, 13], hydrogels [14], magnetically responsive materials [15], or heterogeneous composite approaches [16] that combine dissimilar materials. While these methods can induce folding, they typically suffer from long actuation times, environmental sensitivity, dependence on external fields, and complex multimaterial fabrication, as well as intrinsic interfacial incompatibilities that limit durability and reliable integration, all of which collectively hinder manufacturability and integration.

In this work, we present a mechanically origami‐coded 3D kirigami platform that achieves programmable 3DPVs through a single stretching–release cycle. Unit folding is initiated and propagated along predesigned hinges, rotational axes, and buckling‐control elements, driving full origami activation. During this process, solar cell and interconnect arrays are reconfigured into precise 3D architectures, providing unprecedented design freedom between stretchability and areal coverage. By applying origami codes optimized for ultrastretchability, the system achieves up to 500% system strain while maintaining nearly 100% initial areal coverage. Conversely, with origami codes optimized for ultrahigh energy density, the system maintains more than 100% normalized effective areal coverage (relative to the footprint) even under ∼100% tensile strain.

In the programmable 3DPV system, a nano‐root–anchored AgNWs–In–Ga multiphasic alloy conductor featuring localized AgNW‐In interfacial alloying is developed as an intrinsically integrated electrode. This electrode is structurally co‐designed with the mechanically origami‐coded 3D kirigami platform, enabling stable electrical performance under large mechanical deformation. Although EGaIn and silver can form alloys with high conductivity and mechanical compliance, direct integration of AgNW networks with EGaIn has been hindered by the high surface tension of EGaIn, which disrupts the percolation network. Here, AgNWs are mechanically anchored as nano‐roots on the surface of 3D kirigami, preventing network collapse upon EGaIn application. Within this anchored AgNW mesh network, which retains mechanical compliance under tensile deformation, localized AgNW‐In interfacial alloying is considered to occur within the AgNWs–liquid‐metal network, contributing to a multiphasic AgNWs–In–Ga multiphasic alloy conductive network. This architecture forms a stable composite conductor that combines high conductivity, stretchability, and strong interfacial adhesion. This conductor operates reliably even in ultrastretchable or ultradense origami‐programmed PV configurations, supporting reversible stack‐and‐connect operation.

Collectively, this work establishes a self‐folding 3DPV platform that unites geometric programmability with robust electrical integration in a reversible, single‐material‐based system. By overcoming the intrinsic trade‐off between stretchability and areal coverage, this strategy offers a practical and manufacturable pathway toward programmable freeform PVs and multifunctional 3D energy systems. Beyond static 3DPVs, the platform provides a foundation for next‐generation technologies, including wearable electronics, urban distributed PV, and adaptive electronic devices.

## 2 Results and Discussion

### 2.1 Design and Operating Mechanism of the Mechanically Origami‐Coded 3D Kirigami Platform

#### 2.1.1 Principle of Programmable 3DPV

Three‐dimensional photovoltaics (3DPVs) demonstrate that 3D integration of solar cells can provide a route to increasing effective active‐area deployment and projected power output within a confined footprint. However, most prior 3D implementations have been limited to rigid applications, leaving their potential for stretchable applications largely untapped. Compared to intrinsically stretchable solar cells, the structurally stretchable approach—using rigid islands connected by stretchable interconnects—offers compatibility with existing technologies, as well as improved durability and process compatibility. Yet, this strategy inevitably suffers from a trade‐off between stretchability and areal coverage, since both functional devices and interconnects must share the same confined 2D area. This work addresses these limitations by introducing a mechanically origami‐coded self‐folding 3D kirigami architecture, which enables freeform design and structural reconfiguration to overcome the intrinsic trade‐offs of 2D stretchable systems.

**FIGURE 1:**
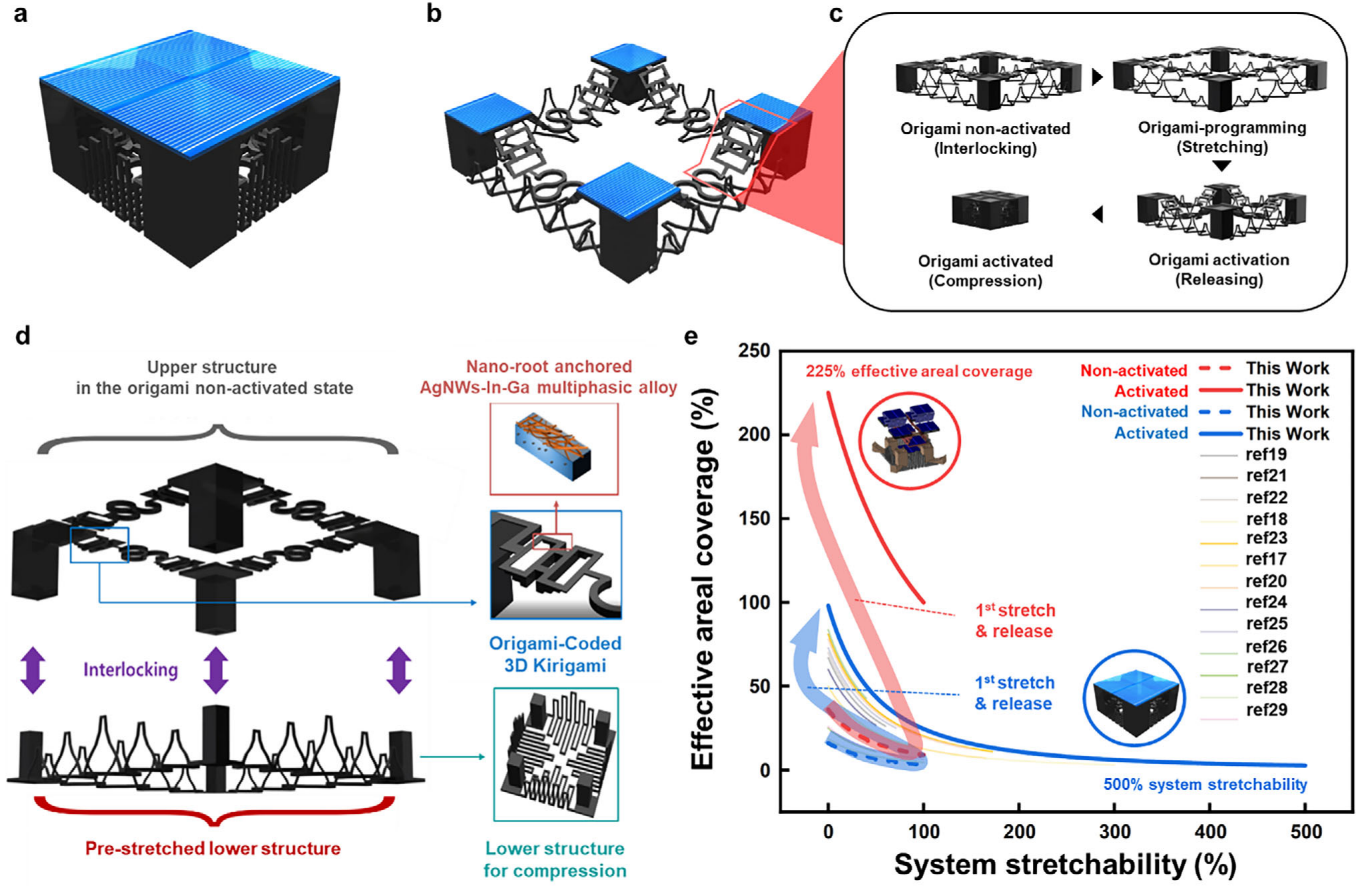
Principle of programmable 3D photovoltaics and performance metrics. (a) Origami‐activated programmable 3D photovoltaics with high areal coverage. (b) Hidden, self‐foldable, stretchable structure enabled by a mechanically origami‐coded 3D kirigami‐based design. (c) Operating mechanism of the mechanically origami‐coded 3D kirigami platform. (d) Components of the mechanically origami‐coded 3D platform for programmable 3D photovoltaics. (e) Comparison of the developed programmable 3D photovoltaic system with previous studies in terms of ultrahigh stretchability (blue) and ultrahigh effective areal coverage (red) [17, 18, 19, 20, 21, 22, 23, 24, 25, 26, 27, 28, 29].

To realize mechanically encoded origami self‐folding, we employed an FDM 3D printer to fabricate the 3D kirigami framework and integrated a nanostructure‐based conductive composite to ensure electrical connectivity. As shown in Figure 1a, the origami‐programmed 3DPV achieves nearly 100% areal coverage in its initial state. Upon stretching the module, previously hidden stretchable conductive interconnects emerge beneath the solar cells (Figure 1b), thereby imparting ultrastretchability to the system. This mechanism directly demonstrates that origami coding can structurally decouple the long‐standing trade‐off between stretchability and areal coverage in 2D stretchable electronics.

The operating mechanism is schematically illustrated in Figure 1c. The upper kirigami structure, which encodes the origami pattern, is interlocked with a lower structure designed to provide restoring force. This configuration forms the origami non‐activated state. Under tensile loading, the kirigami units initiate folding, thereby triggering origami activation. Upon release, the restoring force of the lower structure amplifies the folding process, driving the system toward full origami activation and guiding it along predefined folding pathways.

As summarized in Figure 1d, the platform is composed of three essential elements: (i) the upper structure (origami‐coded kirigami parts), (ii) the lower structure (providing restoring force during origami activation), and (iii) the conductive network (providing current pathways for the PV). The upper structure integrates serpentine features to maximize stretchability and employs thickness modulation to selectively control in‐plane and out‐of‐plane deformation. The lower structure adopts a 3D spring‐like design to provide sufficient restoring force for recovering the folded state of the upper structure, while maintaining a minimal footprint and enabling large stretch range. By stretching the lower structure and interlocking it with the upper structure before folding initiation occurs, an interlocked state prior to origami activation can be assembled.

Despite being fabricated from a single material, this mechanically origami‐coded interlocked 3D kirigami platform leverages geometric design to realize programmable 3D reconfiguration through only a single extension–release cycle. Further details of the platform's components and operation mechanism are provided in Movie S1.

Notably, the upper structure inherently incorporates a nano‐root–anchored AgNWs–In–Ga multiphasic alloy conductor, which remains mechanically compliant while enabling robust electrical connection through simple ohmic contact. Because this conductor can be directly integrated into the substrate through a simple coating process during fabrication, no additional electrode fabrication or wiring steps are required to connect the devices. Consequently, stable electrical connection can be achieved through simple mechanical contact between the 3DPV platform and the solar cells. Furthermore, the conductor simultaneously provides high electrical conductivity and mechanical compliance, ensuring durability, stretchability, flexibility, and reliable electrical connectivity under repeated mechanical deformation.

Leveraging these properties, the platform is fully compatible with photovoltaic devices and thus functions as a programmable 3D photovoltaic system. A direct comparison of performance metrics with prior studies is presented in Figure 1e. Incorporating serpentine elements into the origami‐coded 3D kirigami of the upper structure significantly enhances stretchability (blue). In contrast, replacing the serpentine layout with a rigid‐island design and applying an origami coding strategy tailored for high areal coverage and energy density maximizes areal coverage (red). In the graph, dashed lines denote the stretching process and the corresponding changes in areal coverage from the non‐activated state to the activation point, with arrows indicating the onset of origami activation. Solid lines represent the stretching process and areal coverage after activation.

Collectively, these results demonstrate that origami programming enables structurally encoded 3DPVs to achieve either ultrahigh stretchability (up to 500% system strain while retaining nearly 100% initial areal coverage of active devices) or ultrahigh effective areal coverage (up to 225% while maintaining >100% effective areal coverage during ∼100% system stretching). These metrics substantially outperform conventional stretchable energy systems, establishing origami‐coded 3D kirigami as a powerful enabler for next‐generation deformable photovoltaics.

#### 2.1.2 Working Principle of Mechanically Origami‐Coded 3D Kirigami

**FIGURE 2:**
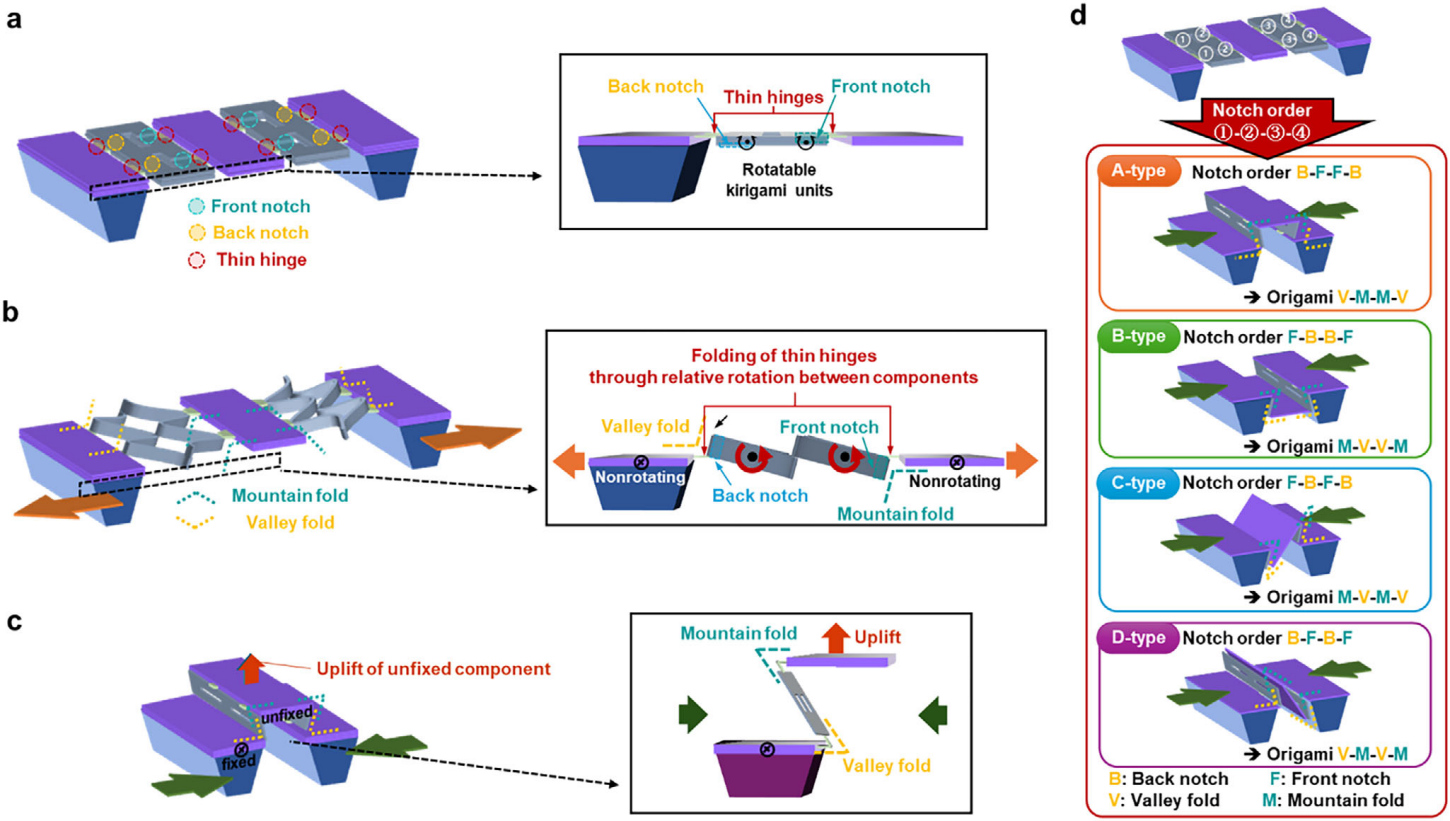
Operating mechanism of mechanically origami‐coded 3D kirigami based on geometric design. (a–c) Origami activation process governed by notch and hinge configuration: (a) initial unit design with encoded front and back notches connected by thin hinges, (b) folding initiation of 3D kirigami units under uniaxial stretching, and (c) full origami activation driven by compression, guiding the structure to fold along predefined pathways. (d) Four distinct origami modes (A–D) determined by different combinations of front‐ and back‐notch designs within the 3D kirigami units, each leading to unique mountain–valley folding patterns.

Kirigami structures become particularly versatile when notches are introduced, as these notches provide an efficient means to control the otherwise unconstrained rotation of kirigami units into predefined orientations [30], an approach that has been reported in the context of mechanical metamaterials and kirigami‐based structures. In this study, we exploit this concept by coupling rotatable 3D kirigami units with non‐rotatable units to induce folding along strategically programmed connections. Specifically, the introduction of front or back notches into rotatable units allows precise control over their rotation direction, while these rotating units are linked to fixed counterparts through thin hinges. Upon stretching, the rotation of the notched units drives hinge‐guided folding, which is subsequently transformed into a fully folded origami structure upon compression. The relative placement of front and back notches dictates the rotation polarity, thereby enabling programmed assignment of mountain or valley folding pathways at the thin hinges. This design strategy thus establishes the conceptual foundation for the programmable mechanisms described in Figure 2 and the following discussion.

In the unit design (Figure 2a), the front and back notches are arranged as a pair within a single origami‐coded 3D kirigami unit. By placing a z‐axis‐free segment between rotatable 3D kirigami segments and constraining both ends with fixed z‐axis segments, a system capable of inducing segmental 3D origami activation can be designed. During uniaxial stretching (Figure 2b), the configuration of the front and back notches determines the rotation direction, either clockwise or counterclockwise, which in turn induces mountain and valley folding through the programmed response of the thin hinges. This stage corresponds to the detailed process of unit folding initiation. Upon subsequent compression (Figure 2c), full origami activation is guided by the pre‐shaped hinges formed during the prior tensile initiation. The key to this system lies in the strategically placed notches and thin hinges, which direct folding pathways along predefined hinge axes. Additionally, thickness modulation enables selective buckling at targeted locations, thereby enhancing the precision of folding and enabling controlled movement along the z‐axis, ultimately achieving directional origami activation. Through this mechanism, the system enables programmable origami activation via simple uniaxial stretching and compression, eliminating the need for complex multidirectional loading. Leveraging this principle, four distinct origami modes (Types A, B, C, and D) can be realized according to specific combinations of front‐ and back‐notch placements, as illustrated in Figure 2d.

Because the present platform was fabricated as a single‐material FDM‐printed system, its actuation behavior in this study was governed primarily by geometric design rather than material variation. However, the layer‐by‐layer deposition process introduces fabrication‐specific characteristics, including local filament morphology, interlayer fusion, and slight geometric rounding at thin features, which can vary with the printing conditions and influence the actual deformation behavior of the printed structures. Accordingly, experimental verification and a systematic empirical design‐window study were prioritized to directly evaluate the actuation behavior of the printed structures and to identify the geometric conditions required for reliable programmed origami activation.

The operation sequence was first outlined using the Type A structure as a representative example. As illustrated in Figure S1, the programmed origami activation mechanism of the kirigami architecture can be understood through the sequential tilting and folding behavior of the structural units. To experimentally verify the rapid and spontaneous programmed origami activation of the fabricated Type A structure, high‐speed camera measurements were conducted. The initiation and activation behavior during deformation was visually confirmed, as shown in Figure S2 and Movie S2.

Based on the experimentally verified structure and operation behavior, the structural design parameters governing the programmed origami activation of the upper 3D kirigami architecture were systematically investigated. To elucidate how the key geometric parameters of the upper structure influence programmed origami operation, the minimum design requirements were experimentally validated through a design‐window approach. The overall structural thickness (Figure S4), slit geometry (Figure S5), notch geometry (Figure S6), and hinge geometry (Figure S7) were treated as the primary design variables of the upper structure and systematically varied. Through this investigation, the minimum geometric conditions required for reliable programmed origami activation were identified within the tested design space. Based on these design windows, additional application‐oriented structural designs were implemented, and the corresponding results are shown in Figure S8.

### 2.2 Combinatorially Programmable Origami‐Coded Arrays and Functional Demonstrations

#### 2.2.1 Versatile and Combinatorially Programmable Origami‐Coded 3D Kirigami Structure

Following the unit‐level control of directional folding demonstrated in Section 2.1, the next logical step is to examine how these origami codes can be extended to multi‐unit assemblies enabled by the combinatorial design space of origami. The true utility of mechanically origami‐coded 3D kirigami lies not only in programming individual units but also in assembling these codes into collective arrays, where complex and application‐specific functionalities emerge from simple uniaxial stretch–compression cycles. By integrating different types of origami units within a single architecture, programmable patterns and structural reconfigurations can be realized at the system level, enabling new possibilities for adaptive and multifunctional devices.

**FIGURE 3:**
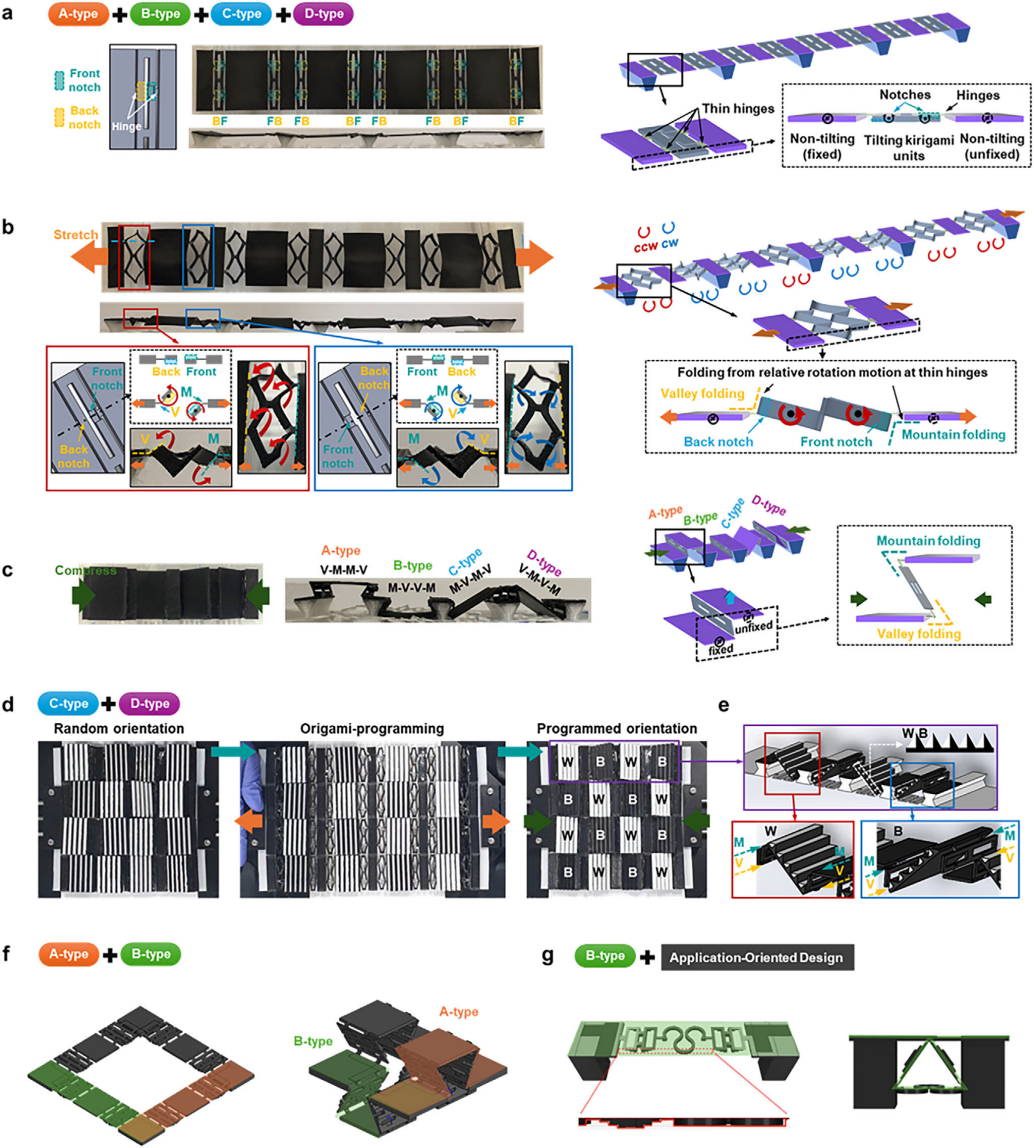
Versatile and combinatorially programmable origami‐coded 3D kirigami arrays. (a–c) Origami operation sequence of a single structure integrating four origami modes (Types A–D): (a) initial configuration, (b) unit folding initiation under uniaxial stretching, and (c) full origami activation after compression. (d,e) Self‐folding chessboard realized by a 4 × 4 array of Types C and D units: (d) operation sequence and (e) unit composition and folding pathways. (f) Programmable origami activation of a 2×2 array combining Types A and B. (g) Application‐specific structural design based on the Type B configuration.

The operation sequence of the four origami types (A, B, C, and D) arranged in a single row is demonstrated in Figure 3a–c, using both real images and schematics of the structures. Four types of origami‐coded 3D kirigami can be integrated into a single structure (Figure 3a), and a single uniaxial stretching step simultaneously triggers unit folding initiation for all structures (Figure 3b). Subsequent uniaxial compression results in full origami activation, yielding four distinct self‐folded configurations (Figure 3c). In the same way that individual origami units respond to a single stretch–release cycle, multiple units can also be programmed and activated using this simple sequence, enabling array‐level implementations for more complex applications.

This concept is further exemplified by the programmable chessboard configuration shown in Figure 3d,e, realized through a 4 × 4 array composed of alternating Type C and Type D units. While the initial state exhibits a seemingly random distribution of black and white patterns, a distinct chessboard pattern emerges after a complete stretch–compression cycle (Figure 3d). Each unit consists of multiple triangular prism structures visually divided into black and white halves. Upon origami folding, Type C units tilt left to reveal the black half, whereas Type D units tilt right to reveal the white half. The combination of programmed origami activation and contrasting coloration results in a clear chessboard pattern (Figure 3e), as demonstrated in Movie S3. Beyond this demonstration, the programmed tilting highlights the potential of such designs for adaptive orientation control and reconfigurable applications, while offering geometric flexibility that may influence effective light‐receiving conditions under varying illumination angles.

Another example is presented in Figure 3f, where a 2 × 2 array composed of Type A and Type B units illustrates the transition from the non‐activated state to the origami‐activated state. This configuration is optimized for efficient sharing of z‐axis space, thereby achieving high areal coverage.

An application‐specific design based on the Type B 3D kirigami configuration is presented in Figure 3g. This structure integrates serpentine elements and employs selective modulation of thickness along the z‐axis to enable localized stiffness control. Upon activation, the serpentine region acts as a stretchable segment during tensile loading and as a structural support during compression‐driven origami folding. This configuration is particularly effective for decoupling the trade‐off between high stretchability and areal coverage.

These results establish 3D kirigami‐based mechanical encoding as a versatile and modular platform for programmable structural and activation designs. Taken together, these demonstrations underscore the versatility and scalability of origami‐coded 3D kirigami for structural programming. To fully harness this mechanical potential in practical photovoltaic systems, however, the platform must be coupled with conductors that can endure localized strain concentrations and maintain stable electrical connectivity. This requirement motivates the development of a new class of nano‐root anchored multiphasic alloy conductors, which will be introduced in the following section.

### 2.3 Fabrication and Characterization of Nano‐Root Anchored AgNWs–In–Ga Multiphasic Alloy Conductors

To ensure reliable electrical performance under repeated and complex mechanical loading, conductors used in origami‐based platforms must combine high electrical conductivity, strong surface adhesion, mechanical durability, and stretchability. A variety of stretchable electrode materials has been explored, including carbon nanomaterials such as CNTs and graphene, metallic nanowires, and liquid metals like EGaIn. In this work, we focus on metallic systems and select AgNWs and EGaIn as representative candidates to secure sufficiently high conductivity for photovoltaic applications. AgNWs provide excellent conductivity and flexibility, delivering superior electrical performance even at low percolation thresholds [31, 32, 33]. However, AgNWs often exhibit limited adhesion to substrates, and establishing reliable electrical networks typically requires additional treatments such as high‐temperature soldering, photolithography, or ion/e‐beam processing, which increase fabrication complexity and cost [34]. Moreover, AgNWs are prone to oxidation, which compromises their long‐term durability. Conversely, EGaIn, owing to its liquid nature, offers intrinsic stretchability and facile electrical connectivity, while also exhibiting a low melting point (15.7°C), high conductivity, and low toxicity [35, 36, 37, 38]. Yet, the spontaneous formation of a Ga_2_O_3_ oxide layer causes uncontrolled wetting and unstable interfacial contact [39], necessitating multiple additional treatments including chemical modification, HCl cleaning, capillary infiltration, biphasic synthesis, conductive polymer composites, and Ag flake ink formulations [40, 41, 42, 43, 44, 45, 46, 47, 48, 49, 50]. While the protective and flow‐regulating effects of the oxide layer [51, 52, 53], the strong reactivity between indium and silver [54, 55, 56, 57], and the capillary action of AgNWs [50] can offer synergistic opportunities, these approaches still rely on post‐treatment such as laser sintering and selective etching, which limit large‐area processing. Furthermore, rather than fully exploiting the intrinsic conductivity of the AgNW network, electrical continuity is often disrupted by aggregation between the liquid metal and AgNWs. These challenges underscore the critical need for a conductor that combines high conductivity, strong adhesion, and mechanical durability while overcoming the intrinsic material limitations of existing systems. A strategy to address these requirements is described below.

#### 2.3.1 Fabrication and Characterization of Nano‐Root Anchored AgNWs–In–Ga Multiphasic Alloy Conductor for 3D Kirigami‐Based Structure

**FIGURE 4:**
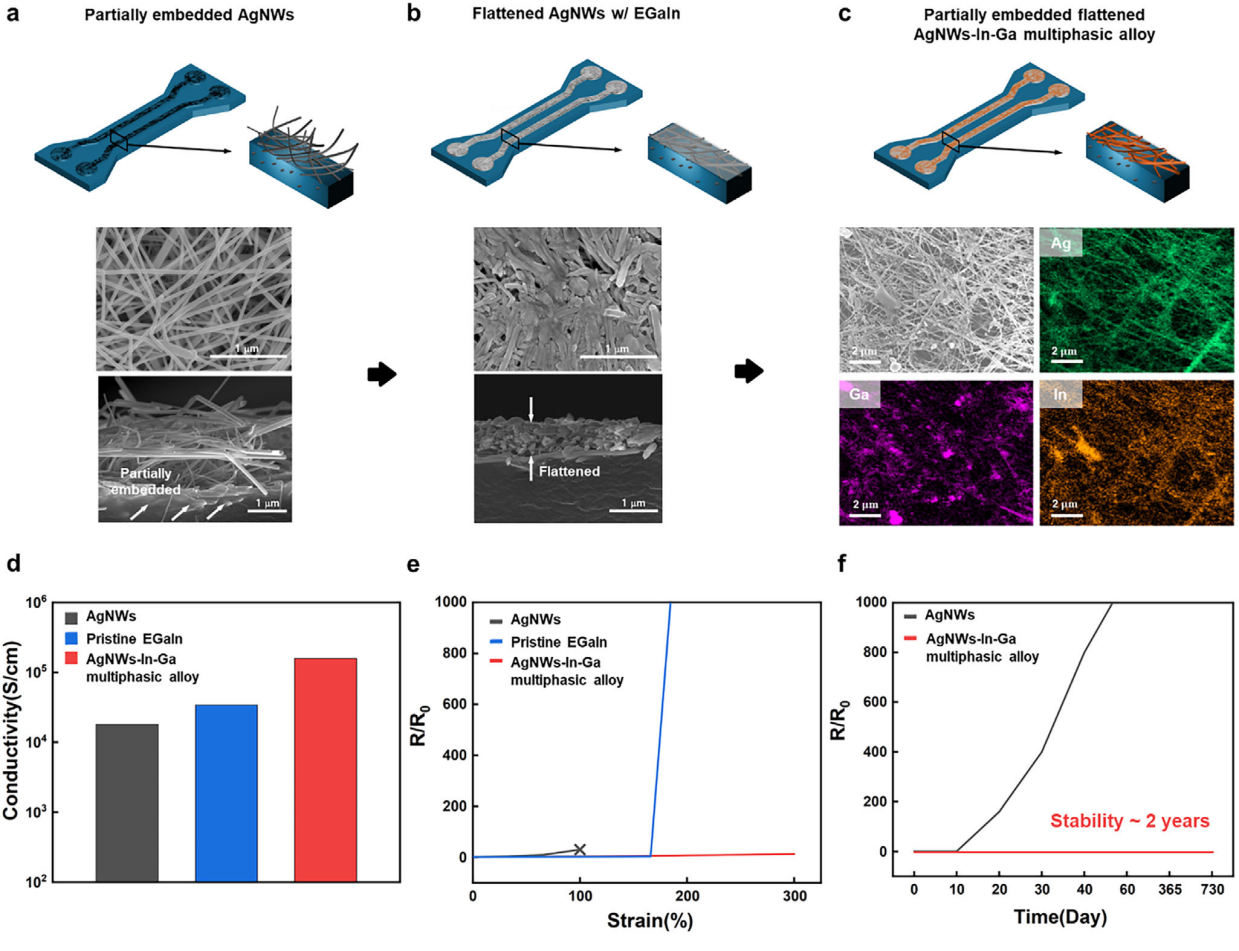
Fabrication steps and comprehensive characterization of the nano‐root anchored AgNWs–In–Ga multiphasic alloy conductor. (a–c) Fabrication process and corresponding SEM and EDS analyses: (a) schematic illustration and surface and cross‐sectional SEM images (scale bars, 1 µm) of the conductor after 3D printing‐based AgNWs transfer; (b) schematic and surface and cross‐sectional SEM images (scale bars, 1 µm) of the conductor after EGaIn application; (c) schematic and EDS elemental maps of the AgNWs–In–Ga multiphasic alloy (Ag, Ga, In; scale bar, 2 µm). (d) Electrical conductivity comparison among AgNWs‐only (gray), pristine EGaIn (blue), and AgNWs–In–Ga multiphasic alloy (red). (e) Relative resistance change under tensile strain for each conductor type: AgNWs‐only (gray), pristine EGaIn (blue), and AgNWs–In–Ga multiphasic alloy (red). (f) Long‐term stability test results under ambient conditions (∼2 years): AgNWs‐only (gray) and AgNWs–In–Ga multiphasic alloy (red).

To enable effective integration with the mechanically origami‐coded 3D kirigami‐based platform, we developed a nano‐root anchored AgNWs–In–Ga multiphasic alloy conductor that is compatible with the structure fabrication process while offering high electrical performance and stretchability. Figure 4a–c summarizes the fabrication steps and the corresponding SEM and EDS analyses.

In the first step, AgNWs are partially embedded into the structure using an FDM‐based 3D printing process to form a nano‐root structure, while bush‐like AgNWs protrude as a structural continuation onto the surface. Surface and cross‐sectional SEM images in Figure 4a confirm the formation of this integrated root‐based, bush‐like AgNW scaffold. This nano‐root architecture strengthens interfacial adhesion to the kirigami surface and serves as a mechanically anchored template for subsequent liquid‐metal integration.

In the second step, EGaIn is applied onto the root‐anchored AgNWs through a mechanical rupturing and rubbing process to form a conformal composite. At this stage, the strong capillary action of the AgNWs and the high surface tension of EGaIn interact synergistically, suppressing local aggregation of EGaIn and AgNWs while promoting flattening and gap filling. Surface and cross‐sectional SEM images in Figure 4b confirm the flattened and conformally integrated morphology of the composite, which enhances the continuity of the percolation network. In contrast, in the absence of the nano‐root structure, aggregation between EGaIn and AgNWs leads to discontinuous electrical pathways, as shown in Figure S9. Notably, this anchored scaffold‐and‐infiltration process is tolerant to moderate variations in the amount of AgNWs or EGaIn, without requiring additional pre‐ or post‐treatments.

Finally, upon EGaIn application, a spontaneous alloying interaction between indium (In) and silver (Ag) occurs upon EGaIn application, resulting in an AgNWs–In–Ga multiphasic alloyed conductive network with a multiphasic character. As shown in the EDS elemental mappings in Figure 4c, indium is preferentially distributed along the AgNW pathways, while gallium forms a continuous phase that bridges the indium‐enriched regions. These observations indicate that the conductor functions not merely as a physical mixture, but as an integrated multiphasic alloy network featuring alloy‐based conductive pathways. Figure S10 further confirms that this alloying behavior occurs consistently across different AgNW loadings. The detailed fabrication process, including the transfer, flattening, and alloying steps, is illustrated in Figure S11 and visualized through a 3D animation in Movie S4.

To further investigate the interfacial interactions, additional cross‐sectional line‐scan analyses were performed. The results show that indium exhibits correlated distributions with both Ag‐rich and Ga‐rich regions, suggesting that indium remains associated with both the Ag interface and the surrounding Ga–In matrix rather than being fully consumed by AgNW–In reactions (Figure S12).

In addition, EDS line‐scan analysis conducted after acetone rinsing shows that the spatial distributions of Ga, In, and Ag maintain concentration peak patterns similar to those observed prior to rinsing. This observation suggests that the elemental distributions are not simply attributable to residual liquid metal but are consistent with interfacial interactions at the AgNWs–liquid metal interface.

Furthermore, high‐magnification SEM observations show that the nanowire mesh network structure remains preserved after removal of excess liquid metal, maintaining its mechanically compliant conductive framework (Figure S13).

The nano‐root anchored AgNWs–In–Ga multiphasic alloy conductor fabricated as described above achieves a high electrical conductivity of 157 600 S/cm (Figure 4d). This performance arises from the inherently integrated structure and electrically continuous pathways enabled by synergistic interactions between AgNWs and pristine EGaIn, which are otherwise unattainable by either component alone. Notably, the AgNW network is bound by the highly fluid EGaIn, imparting excellent intrinsic stretchability. The multiphasic alloy conductor maintains a stable resistance response even under tensile strain (Figure 4e), in contrast to the collapse of conduction pathways in AgNWs‐only conductors or the local aggregation and resistance increase induced by volumetric changes and high surface tension in pristine EGaIn. The measurement configuration is described in Figure S14.

Furthermore, a native Ga_2_O_3_ oxide layer spontaneously forms from exposed gallium at the composite surface. This oxide is chemically and thermally stable and has been widely studied in semiconductor and power‐device contexts [58, 59]. In the present composite system, the oxide forms in a self‐limiting manner at the air interface and is expected to act as a passivating barrier that limits environmental interaction with the underlying AgNWs–In–Ga conductive network. In addition, the origami‐coded structural design distributes mechanical deformation across programmed hinge regions, thereby avoiding sustained tensile strain in the interconnects and mitigating cumulative degradation.

Consistent with this interpretation, no measurable electrical degradation was observed during >2 years of indoor ambient storage despite seasonal environmental variations, including humid summer and low‐temperature winter conditions (Figure 4f). To further assess environmental robustness, additional resistance measurements were conducted over a temperature range of −20 to +50°C. Negligible resistance variation was observed throughout this range (Figure S15), indicating stable electrical performance under temperature variation.

In addition to environmental stability, the conductor also exhibits robust electromechanical performance. Relative resistance changes were monitored under repeated folding and underwater operation for 10 000 cycles each, demonstrating stable electrical behavior (Figures S16 and S17). Stable electrical contact is also maintained during rotation and across different orientations with respect to gravity through simple ohmic contact between facing conductors (Figure S18). This behavior is consistent with localized interfacial alloying within the AgNW network, which is considered to contribute to the formation of a mechanically compliant alloy conductor. Movie S5 further demonstrates stable LED operation in both air and underwater environments via direct ohmic contact between facing conductors, without any adhesive or additional conductor processing.

Collectively, this composite conductor spontaneously forms a stretchable and conductive alloyed network together with a surface passivation layer (Ga_2_O_3_). While this layer typically acts as a protective coating, it ruptures upon external physical contact to provide electrical connectivity, achieved solely through AgNW transfer and EGaIn application without complex post‐treatment or additional fabrication steps. Furthermore, preferential accumulation of indium around silver induces In‐rich and Ga‐rich regions with local compositions deviating from eutectic Ga–In, imparting an intrinsic biphasic nature that further enhances adaptability and functionality.

Through nano‐root anchoring, AgNWs overcome their intrinsic incompatibility with EGaIn, enabling seamless integration into a unified conductor. As a result, the conductor simultaneously achieves the high conductivity of AgNWs and the intrinsic stretchability of EGaIn while eliminating the need for additional processing. This spontaneous alloying and structural anchoring strategy establishes a durable and multifunctional conductor that transcends the fundamental limitations of its base materials. Moreover, owing to its process‐level compatibility with 3D kirigami fabrication, the conductor can be co‐fabricated and seamlessly integrated into origami‐coded platforms, enabling reversible stack‐and‐connect modules that maintain stable ohmic interconnections and mechanical functionality.

### 2.4 Reversible Stack‐and‐Connect Programmable Photovoltaics

#### 2.4.1 Reversible Stack‐and‐Connect Origami‐Coded 3D Kirigami Platform With the Nano‐Root Anchored AgNWs–In–Ga Multiphasic Alloy

With the nano‐root anchored AgNWs–In–Ga multiphasic alloy conductor integrated into the mechanically origami‐coded 3D kirigami platform (Section 2.3), we present representative demonstrations illustrating how the conductor can support reconfigurable device interfacing on the platform. For adaptive photovoltaic systems, it is beneficial to achieve not only mechanical resilience and electrical stability under deformation, but also the ability to reversibly attach, detach, and reconfigure functional modules without complex wiring or permanent bonding. The stack‐and‐connect concept explored here illustrates the feasibility of using ohmic contact alone to form practical interconnections across representative devices, while enabling flexible reconfiguration through simple mechanical stacking.

**FIGURE 5:**
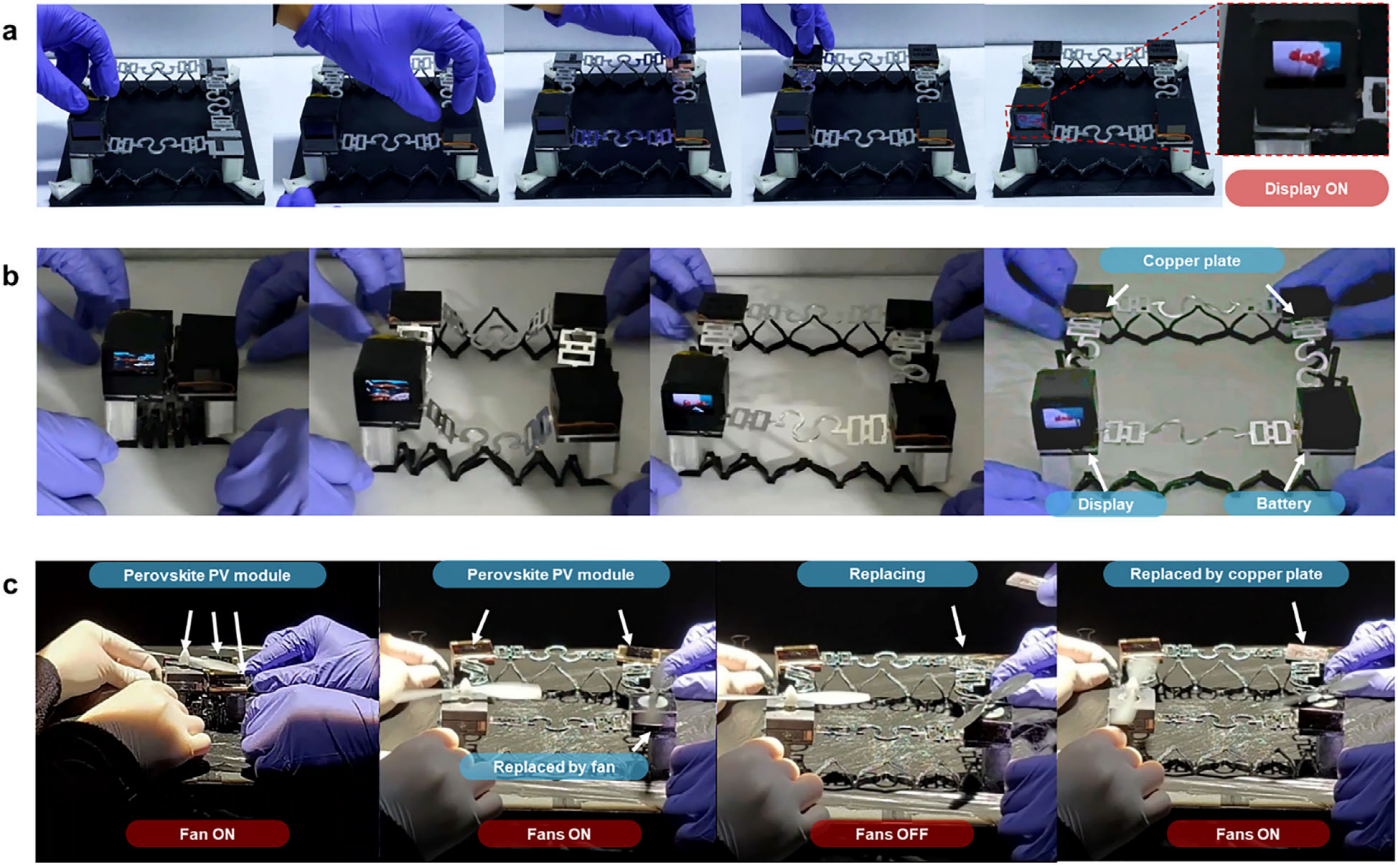
Demonstrations of reversible stack‐and‐connect operation on the origami‐coded 3D kirigami platform. (a) Display operation enabled by ohmic‐contact‐based electrical integration. (b) Stable display functionality under stretching and compression. (c) Fan operation enabled by reversible stack‐and‐connect reconfiguration using perovskite PV modules, copper plates, and fans.

This capability is first demonstrated by sequentially stacking a display module, a battery, and a copper‐taped substrate on the platform (Figure 5a). The stable operation of the display confirms that reliable electrical interconnection can be achieved solely through ohmic contact. Importantly, the electrical interface remains functional even while the platform undergoes stretching and compression (Figure 5b), indicating that the contact‐based interconnection is maintained under mechanical deformation. Furthermore, the electrode unit maintains stable electrical performance even after 10 000 tensile cycles under twisted configurations (Figure S19). In addition, the platform preserves stable electrical characteristics after 1000 cycles of repeated deformation on curved surfaces under off‐axis loading conditions (Figure S20).

The reconfigurable nature of the approach is further illustrated using perovskite photovoltaic (PV) modules (Figure 5c). In the compressed state, three PV modules power a single fan. Upon stretching, one PV module is replaced with an additional fan, allowing the remaining two PV modules to drive two fans simultaneously. Removing one module interrupts the circuit and stops fan operation; inserting a copper plate then re‐establishes the electrical pathway, enabling one PV module together with the copper plate to power two fans again. This sequence demonstrates reversible stack‐and‐connect operation, where electrical functionality can be maintained and reconfigured through controlled attachment/detachment of modules via ohmic contact.

Taken together, these demonstrations show that the platform can electrically interface with diverse device modules while allowing reversible replacement or detachment depending on application needs. A detailed demonstration is provided in Movie S6, and the specifications of the devices used are summarized in Figure S21. Based on these demonstrations, we further develop the platform into programmable 3D photovoltaics using commercial silicon solar modules, implementing two operating modes—one emphasizing ultrahigh areal coverage and the other ultrahigh stretchability—as detailed in the following section.

### 2.5 Performance of Programmable 3D Photovoltaics With Extreme Stretchability and Areal Coverage

#### 2.5.1 Programmable 3DPV for Ultrahigh Stretchability and Ultrahigh Effective Areal Coverage

Extending beyond proof‐of‐concept demonstrations, we quantitatively evaluate the photovoltaic performance of the platform, focusing on how the integration of mechanically origami‐coded 3D kirigami and the nano‐root anchored AgNWs–In–Ga multiphasic alloy conductor enables two distinct operating modes, ultrahigh stretchability and ultrahigh areal coverage, that directly address the conventional trade‐off between mechanical deformability and power generation efficiency.

**FIGURE 6:**
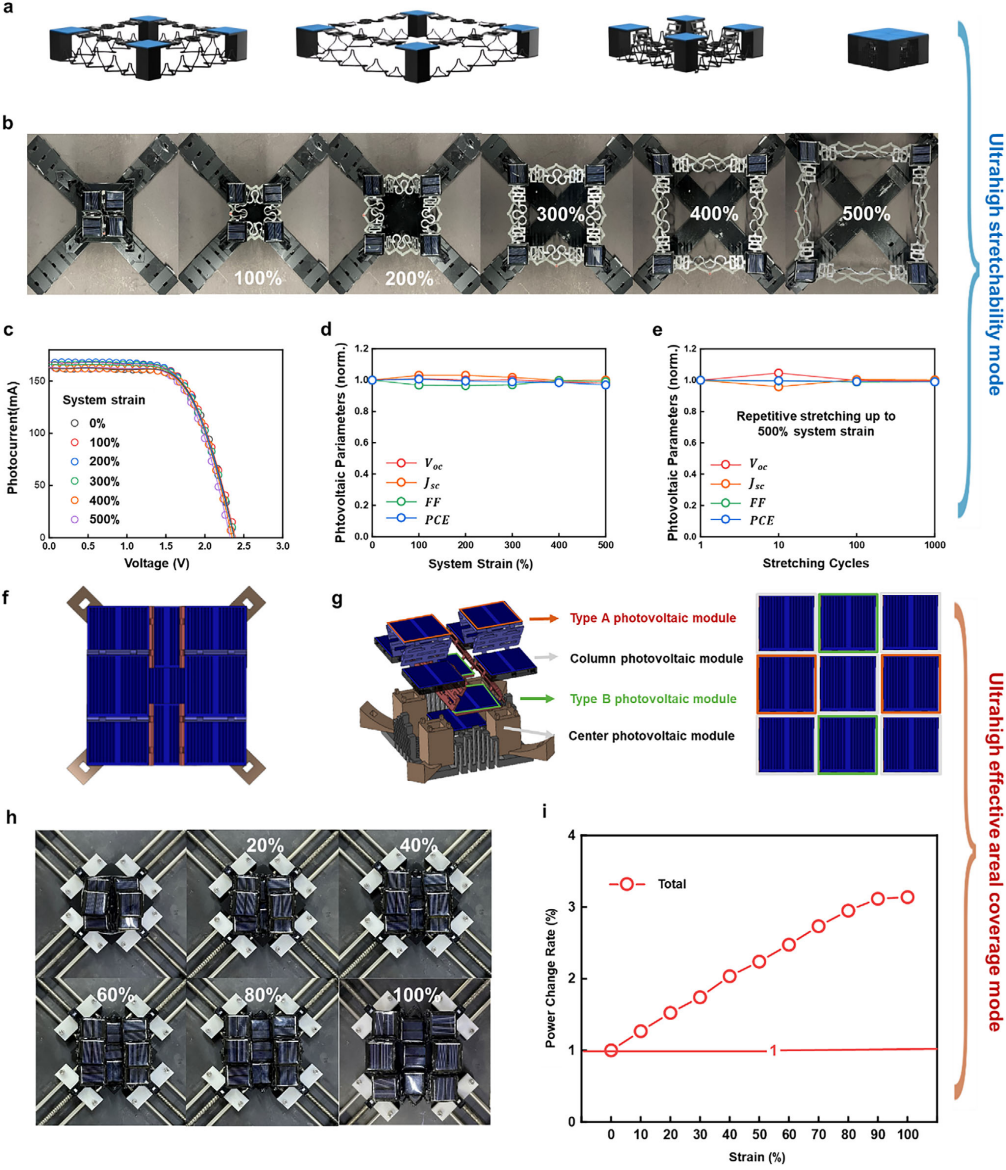
Performance of programmable 3D photovoltaics under ultrahigh stretchability and ultrahigh areal coverage modes. (a‐e) ultrahigh stretchability mode: (a) Programmable origami operation sequence in ultrahigh stretchability mode. (b) Actual stretching of ultrahigh‐stretchable 3D photovoltaics. (c) I–V curves at different strain levels. (d) FF, Voc, Isc, and PCE variations with strain. (e) FF, Voc, Isc, and PCE variations over repeated stretching–recovery cycles(500% system strain). (f‐i) ultrahigh areal coverage mode: (f) Top view of ultrahigh areal coverage 3D photovoltaics. (g) Schematic of ultrahigh effective areal coverage 3D photovoltaics showing the 3D arrangement of photovoltaic modules. (h) Actual stretching of ultrahigh effective areal coverage 3D photovoltaics(100% system strain). (i) Power output variation with strain in ultrahigh effective areal coverage mode.

In the ultrahigh stretchability mode, the origami operation sequence in Figure 6a shows how additional extension of the system initiates unit folding. A top‐view image of the stretching process in Figure 6b clearly visualizes the progressive strain up to 500%. Importantly, the I–V characteristics, including open‐circuit voltage (Voc), short‐circuit current (Isc), fill factor (FF), and power conversion efficiency (PCE), remain nearly unchanged across different strain levels, as demonstrated in Figure 6c,d. Stable photovoltaic performance is preserved even after 1000 repeated stretching–recovery cycles at 500% system strain, as shown in Figure 6e.

In parallel, the ultrahigh areal coverage mode is realized when photovoltaic units replace part of the stretchable serpentine structure used in the ultrahigh stretchability mode, employing both Type A and Type B origami‐coded 3D kirigami units. The array in its origami‐activated state is summarized in Figure 6f, and the corresponding 3D arrangement of photovoltaic modules is illustrated in Figure 6g. The stretching process over a system strain range of 0–100% in Figure 6h confirms that the array achieves an initial areal coverage of up to 225% (relative to the initial projected footprint) while still maintaining full coverage even at 100% system strain. Accordingly, the power output progressively increases with strain, validating the effectiveness of the ultrahigh areal coverage configuration, as the projected area is geometrically reconfigured during deformation (Figure 6i). This behavior illustrates that the platform enables dynamic redistribution of active area without sacrificing mechanical compliance, thereby demonstrating transient mitigation of the stretchability–areal coverage trade‐off.

The measurement setups, procedures, and evaluation criteria used to quantify stretchability and areal coverage are described in Figures S22 and S23, respectively. The maximum achievable system strain (500%) and effective areal coverage (225%) originate from the structural design principle of the lower substructure, which defines the accessible 3D spatial envelope derived from the initial footprint area and the associated geometric constraints. A detailed explanation of this design principle is provided in Figure S24.

Collectively, these results demonstrate that integration of the mechanically origami‐coded 3D kirigami structure with an intrinsically integrated nano‐root anchored AgNWs–In–Ga multiphasic alloy conductor enables a programmable 3D photovoltaic system that maintains excellent electrical and mechanical stability under diverse loading conditions, while simultaneously achieving ultrahigh stretchability and maximized effective areal coverage. Beyond the geometric increase in power output enabled by expanded areal coverage, this decoupling of the conventional trade‐off between stretchability and areal coverage offers critical advantages, establishing a pathway toward durable and multifunctional 3D energy systems.

## 3 Conclusions

In this work, we developed a mechanically origami‐coded 3D kirigami platform that enables programmable 3D photovoltaics (3DPVs) through a simple extension–release sequence. Notch‐ and hinge‐guided geometric encoding converts uniaxial stretching into deterministic unit folding initiation and subsequent full origami activation, enabling precise and repeatable formation of functional 3D architectures without complex multidirectional loading or stimulus‐responsive actuation materials. By implementing distinct origami codes and together with tailored device–interconnect layouts, we realized two representative 3DPV designs targeting opposite performance metrics: an ultrastretchable configuration reaching up to 500% system strain while retaining nearly 100% initial areal coverage of active devices and stable output over 1000 stretching–recovery cycles, and a high‐density configuration achieving up to 225% initial effective areal coverage (relative to the initial projected footprint) while maintaining ≥100% effective areal coverage during ∼100% system stretching. To the best of our knowledge, these results are among the highest reported levels of system stretchability and initial effective areal coverage in rigid‐island–interconnect‐based structurally stretchable electronic/energy‐device architectures, as benchmarked against the studies surveyed in Figure 1e.

Reliable electrical operation under severe folding and stretching is enabled by an intrinsically integrated nano‐root anchored AgNWs–In–Ga multiphasic alloy conductor, in which the mechanically anchored AgNW network preserves electrical continuity during deformation while also supporting reversible stack‐and‐connect interfacing via simple ohmic contact. Overall, the combination of geometric programmability and robust electrical integration provides a practical design route toward freeform, reconfigurable 3D energy platforms, and suggests broader relevance to adaptive electromechanical systems requiring reversible 3D reconfiguration together with stable electrical interfacing.

## 4 Experimental Section

### 4.1 Materials for Nano‐Root Anchored AgNWs–In‐Ga Multiphasic Alloy Conductor Fabrication

Glass substrates (300 mm × 300 mm × 1 mm; Daihan Scientific Group), a silver nanowire (AgNW) dispersion in ethanol (A70, 5 mg mL^−^^1^; Novarials), and eutectic gallium–indium (EGaIn, Ga 75.5%/In 24.5%, ≥99.99% trace metals basis; Merck, Inc., USA), used as received, were used.

### 4.2 Fabrication of the Nano‐Root Anchored AgNWs–In–Ga Multiphasic Alloy Conductor

AgNWs were first coated onto glass substrates using a four‐sided film applicator (Model 1107; SI, UK; gap, 60 µm) mounted on an automatic bar coater (SB‐1000VH; Woomyung Inc., Korea). The substrates were secured on a vacuum chuck, and the coating was performed twice (top‐to‐bottom and bottom‐to‐top directions) at a substrate stage temperature of 60°C and a coating speed of 30 mm s^−1^. The AgNW‐coated glass substrate was then placed on an FDM 3D printer (Style NEO‐A31C; CUBICON Inc., Korea), and TPU was printed directly onto the coated surface (nozzle temperature, 230°C; build‐plate temperature, 60°C; printing speed, 50 mm s^−1^). After printing, the TPU/AgNWs film was detached from the glass substrate, and approximately 0.1 mL of EGaIn was dispensed using a 1 mL syringe and spread over the surface by mechanical rupturing (rubbing) to complete the composite conductor.

### 4.3 Materials for Perovskite Module Fabrication

Cesium iodide (CsI), dimethylformamide (DMF), dimethyl sulfoxide (DMSO), chlorobenzene, isopropyl alcohol, lithium bis(trifluoromethanesulfonyl)imide (Li‐TFSI), and 4‐tert‐butylpyridine (tBP) were purchased from Sigma‐Aldrich. Formamidinium iodide (FAI), methylammonium bromide (MABr), and tris(2‐(1H‐pyrazol‐1‐yl)‐4‐tert‐butylpyridine)cobalt(III) tris(bis(trifluoromethylsulfonyl)imide) (Co(III) TFSI) were purchased from GreatCell Solar Materials. Lead iodide (PbI_2_) and lead bromide (PbBr_2_) were purchased from TCI Korea. A colloidal SnO_2_ solution (15 wt% in water) was purchased from Alfa Aesar. 2,2′,7,7′‐Tetrakis(N,N‐di‐p‐methoxyphenylamino)‐9,9′‐spirobifluorene (Spiro‐OMeTAD, LT‐S922) was purchased from Lumtec.

### 4.4 Fabrication of the Perovskite Solar Module

Etched ITO substrates (10 Ω sq^−^^1^, AMG Tech) were sequentially cleaned with detergent, deionized water, and isopropyl alcohol (15 min each), followed by UV–ozone treatment (20 min). A SnO_2_ colloidal solution was spin‐coated at 3000 rpm for 30 s and annealed at 100°C for 30 min. The perovskite layer, Cs_0_._05_(FA_0_._83_MA_0_._17_)_0_._95_Pb(I_0_._83_Br_0_._17_)_3_ in DMF:DMSO (4:1, v/v), was deposited by a two‐step spin‐coating process (1000 rpm for 10 s; 4000 rpm for 30 s); during the second step, 200 µL of chlorobenzene was dripped 10 s after the start of the step, followed by annealing at 100°C for 60 min. After cooling, Spiro‐OMeTAD in chlorobenzene, doped with Li‐TFSI, Co(III) TFSI, and tBP, was spin‐coated at 4000 rpm for 30 s. An 80 nm Au electrode was then thermally evaporated. For rigid perovskite mini‐modules (3 × 3 cm^2^ ITO substrates), four serially connected cells (2 × 2 cm^2^ aperture, 5 mm length) were patterned. P1 and P2 scribes were performed using a nanosecond laser (KORTherm Science, 1064 nm, 20 MHz, 40 ms) at 30.0% and 11.5% laser power, respectively, and P3 was mechanically scribed to isolate the back contact. Figure S25 shows the structure and photograph of the completed perovskite module.

### 4.5 Electrode Surface Morphology and Elemental Analysis

The electrode surface morphology was characterized by scanning electron microscopy (SEM; Inspect F), and elemental distributions were obtained by energy‐dispersive X‐ray spectroscopy (EDS) mapping.

### 4.6 PCE Measurement

Photovoltaic performance was measured using a source measurement unit (Keithley 2400) and a solar simulator equipped with a 1600 W xenon lamp (Yamashita Denso, YSS‐200A). The light intensity was calibrated to 100 mW cm^−^^2^ (AM 1.5G) using a certified Si reference cell (National Renewable Energy Laboratory, NREL). For demonstrations, a commercial silicon solar mini module (30 × 30 mm^2^; *V*_oc_ = 1.2 V, *I*_sc_ = 80 mA) was employed.

### 4.7 Electrical Characterization

All specimens were fabricated using TPU, and electrical resistance was measured using a digital multimeter (Fluke 179). The following tests were performed. *(i) Stretchability test*: Electrodes with dimensions of 0.2 mm (thickness), 0.4 mm (width), and 30 mm (length) were subjected to tensile testing. The specimens were mounted on a displacement‐controlled tensile stage and stretched along the longitudinal direction. Electrical resistance was monitored during tensile deformation through copper tape connections attached to the electrodes. The specimens were mechanically fixed using a crank‐driven clamping system, which simultaneously provided stable mechanical fixation and electrical ohmic contact. *(ii) Lifetime test*: Electrodes were stored under typical indoor ambient conditions for two years, and their electrical resistance was periodically measured to evaluate long‐term stability. *(iii) Conductivity measurement*: Electrical conductivity was measured using a four‐point probe system (Loresta‐GX MCP‐T700, Nittoseiko Analytech, Japan). The thickness of the electrodes was determined from cross‐sectional SEM observations. *(iv) Rotation test*: Rod‐shaped electrodes with an electrically disconnected center region were fabricated. Electrical continuity between the two ends was restored by attaching a 205.9 mg block carrying an identical electrode pattern. The block was rotated in 30° increments, and the electrical resistance between the two ends was measured using a multimeter connected through external wiring. *(v) Folding test*: Electrodes incorporating a foldable hinge structure were subjected to cyclic folding tests up to 10 000 cycles. The specimens (120 mm × 30 mm × 1 mm) contained a folding notch with a width of 3 mm. Folding motion was generated using a rotational‐to‐sliding conversion mechanism driven by a motorized drill. The system employed a circular disk with a diameter of 150 mm (radius = 75 mm), producing a reciprocating displacement of 150 mm per rotation. At an operating speed of 180 rpm, the average sliding velocity was approximately 450 mm s^−^^1^. The specimens were clamped using a crank‐driven fixing stage connected to copper tape leads, ensuring simultaneous mechanical fixation and stable electrical ohmic contact during the measurement. Resistance changes were measured under both ambient and underwater conditions.

### 4.8 Test for Reversible Stack‐And‐Connect Characteristics

The reversible stack‐and‐connect capability was evaluated using an in‐house‐fabricated perovskite solar module, an OLED display (uOLED‐96‐G2, 0.96‐inch OLED Pack for Arduino, 4D Systems Inc., Australia), a battery (PD2450, 3.7 V, 200 mAh, 5.2 × 24.5 mm, Korea PowerCell Inc., Korea), and a mini DC motor‐driven fan. A metal plate laminated with copper tape (Copper EMI Shielding Tape 1182, 3 M Inc., USA) was used as a conductive bridge to connect segmented electrode patterns. To improve contact reliability, the display, battery, and fan were assembled using planar adapter packages.

## Conflicts of Interest

The authors declare no conflicts of interest.

## Supporting information




**Supporting File**: adma73188‐sup‐0001‐SuppMat.docx.

**Supporting Movie**: adma73188‐sup‐0002‐MovieS1‐S6.zip.

## Data Availability

The data that support the findings of this study are available in the supplementary material of this article.
