# Supplementary material for: Programmable 3D Photovoltaics via Mechanically Origami‐Coded Interlocked 3D Kirigami and Nano‐Root Anchored AgNWs–In–Ga Multiphasic Alloy Conductor
Source: Adv Mater. 2026 Jun 9;38(39):e23685. doi: 10.1002/adma.202523685 (PMC13361253; doi:10.1002/adma.202523685)
Supplement: Supplementary file 1 — Supporting File: adma73188‐sup‐0001‐SuppMat.docx. [file ADMA-38-e23685-s002.docx]

Supporting Information

Programmable 3D Photovoltaics via Mechanically Origami-Coded Interlocked 3D Kirigami and Nano-Root Anchored AgNWs-In-Ga Multiphasic Alloy

Seok Joon Hwang, Jiwon Ryu, Byungsoo Kang, Injong Oh, Dae-Hee Cho, YoungHoi Cho, Seung S. Lee, Dong Hoe Kim, Deokjae Choi, Gee Yeong Kim, Heesuk Jung, Taehee Kim, Hyeonggeun Yu, Seungjun Chung*, Byoung Koun Min*, and Phillip Lee*

**
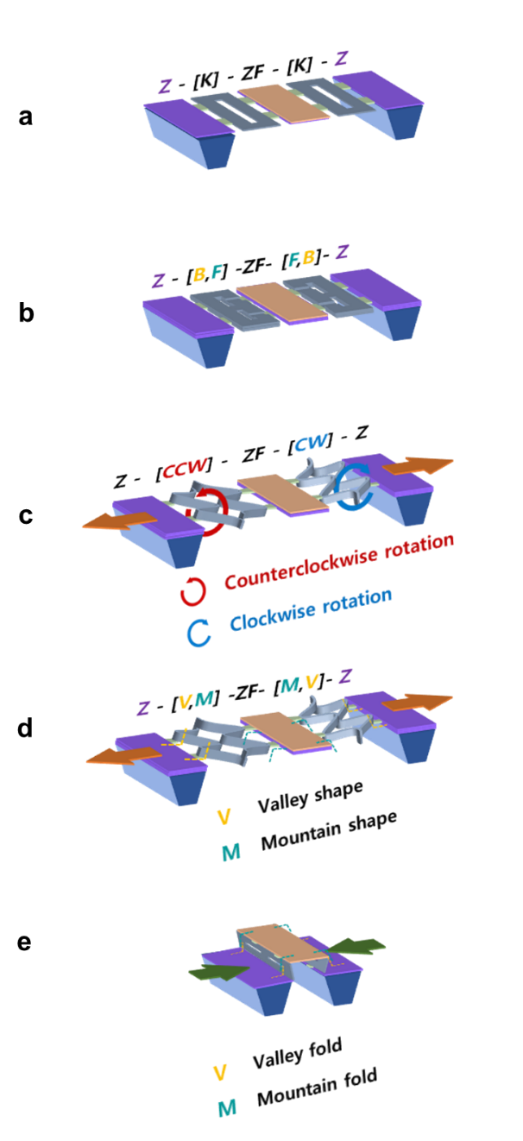
**

**Figure S1.** Schematic illustration of the operation sequence of Type A.

(a) Uncoded unit layout consisting of fixed-z end segments (Z), a central z-free segment (ZF), and two rotatable kirigami segments ([K]), connected by thin hinges (denoted by “–”), represented as Z–[K]–ZF–[K]–Z.

(b) Type A notch coding, where the left rotatable segment is programmed with a back-notch/front-notch ([B,F]) and the right segment with a front-notch/back-notch ([F,B]), represented as Z–[B,F]–ZF–[F,B]–Z.

(c) Unit folding initiation under uniaxial stretching: out-of-plane buckling induces deterministic rotations of the notched kirigami segments, where [B,F] rotates counterclockwise (CCW) and [F,B] rotates clockwise (CW), represented as Z–[CCW]–ZF–[CW]–Z.

(d) The rotations initiate programmed folding at the hinge connections to the non-rotating Z and ZF segments, yielding valley (V) and mountain (M) folds (side view), represented as Z–[V,M]–ZF–[M,V]–Z.

(e) Strain localizes at the thin hinges, pre-shaping the programmed M/V folding geometry. Subsequent compression drives full origami activation into the Type A configuration.

**
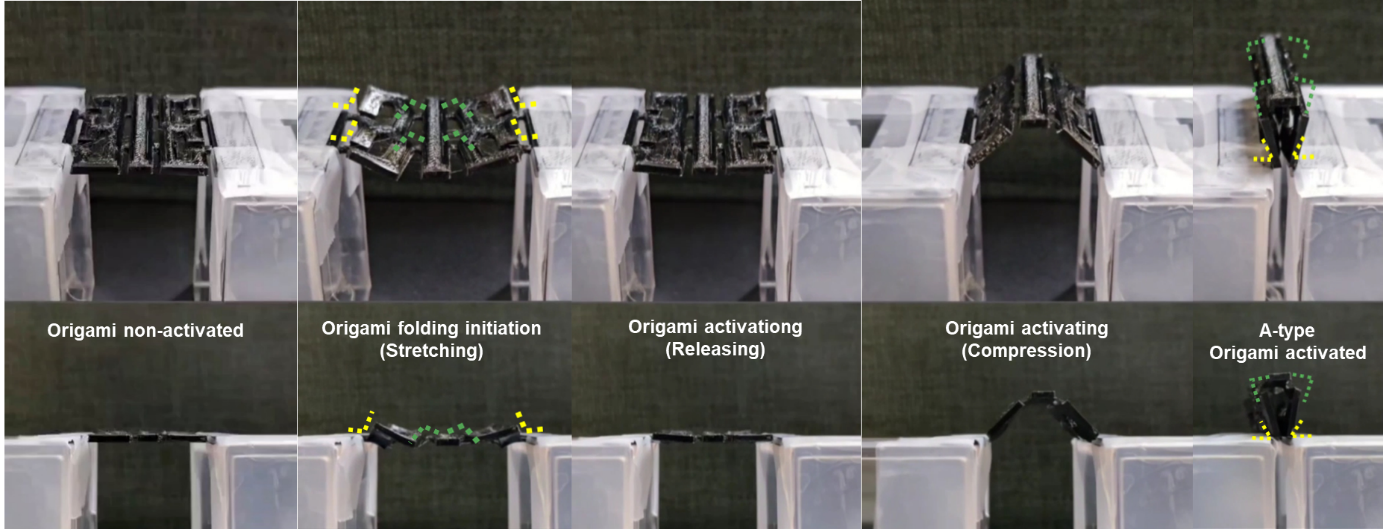
**

**Figure S2.** High-speed camera snapshots of the Type A origami sequence during uniaxial stretching and subsequent compression, showing the transition from the origami non-activated state to the fully origami-activated state.

**
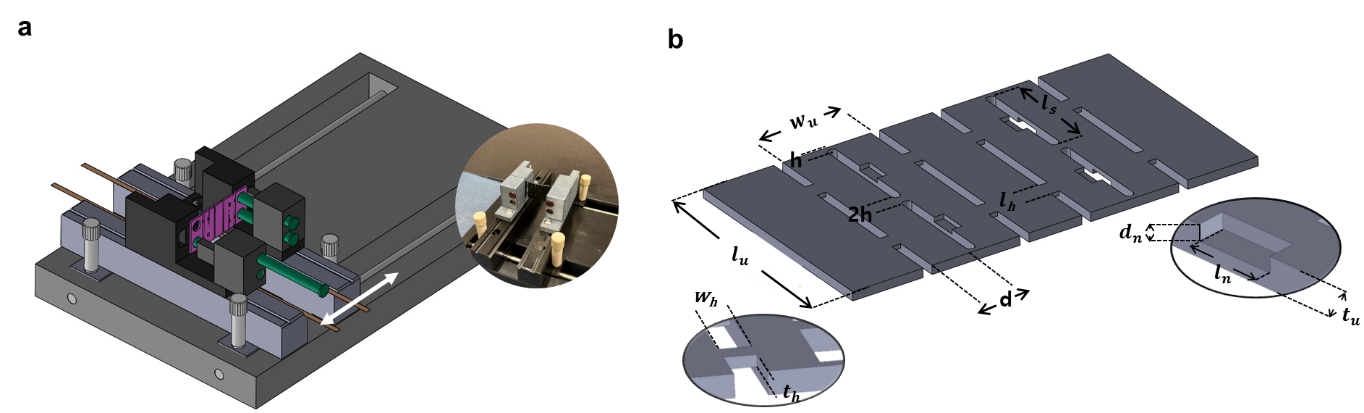
**

**Figure S3.** Mechanical testing setup and geometric parameters for experimental verification of the design window.

(a) Three-dimensional schematic of the mechanical testing setup used to evaluate kirigami-based origami actuation under tension–compression loading, corresponding to the actual measurement setup

(b) Definition of the geometric design parameters used for the experimental verification of the design window. *(* $l_{u}$ = 20 mm, $w_{u}$ = 8 mm, $t_{u}$ = 1 mm, $l_{s}$ = 9 mm, $d$ = 3.9 mm, $h$ = 0.5 mm, $w_{h}$ = 1 mm, $l_{h}$ = 2 mm, $t_{h}$ = 0.5 mm $l_{n}$ = 1.5 mm, $d_{n}$ = 0.5 mm)

**
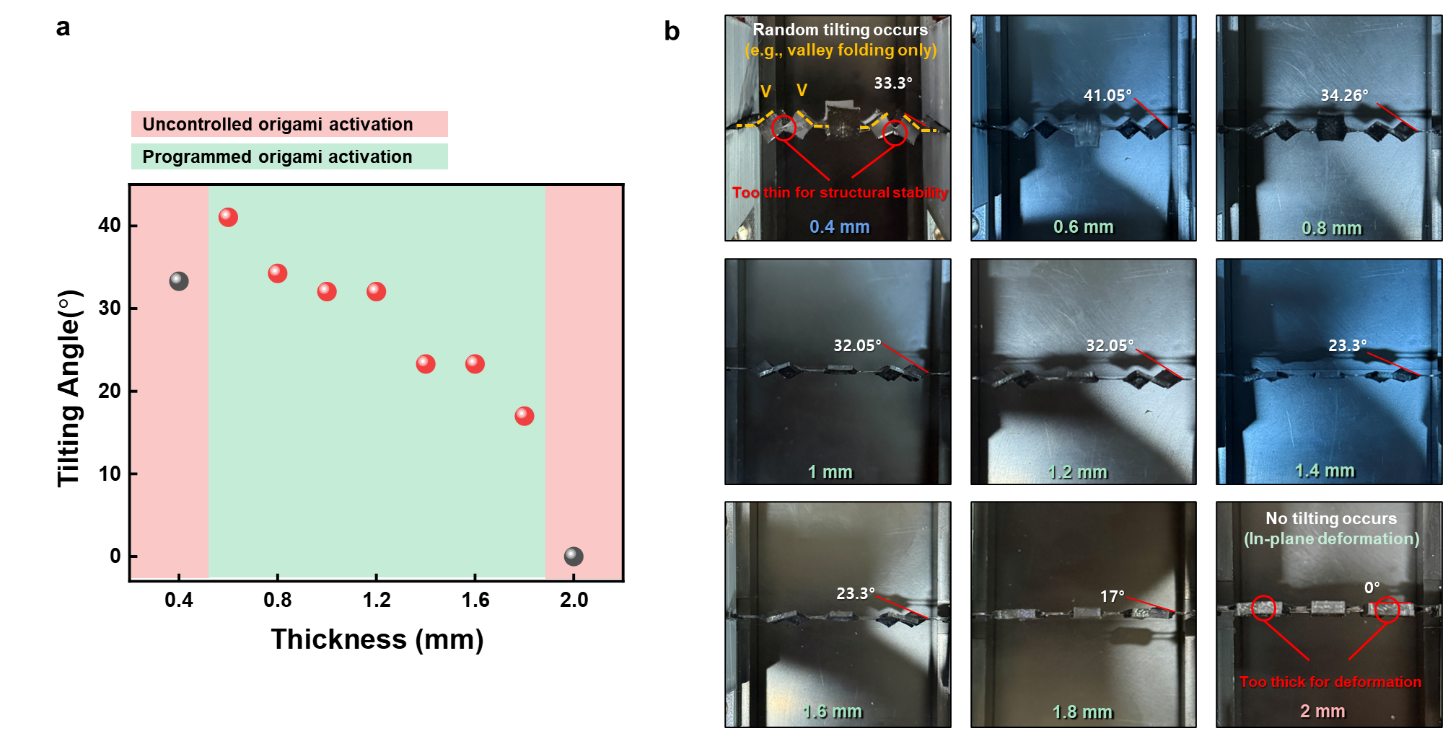
**

**Figure S4.** Design window for structural thickness enabling programmed origami activation:

(a) Graph showing the thickness range in which programmed origami activation can occur. For each condition, two samples were fabricated and tested for 50 repeated cycles. Green regions indicate conditions where the intended origami activation mode was consistently achieved throughout all cycles, whereas red regions denote conditions where the activation behavior was inconsistent.

(b) Tensile deformation behaviors observed at different structural thicknesses.

Considering the minimum printing resolution of the 3D printing process (0.2 mm), the minimum structural thickness required to realize the notch and hinge features was approximately 0.4 mm. To maintain geometric consistency, the design ratios were fixed while the overall structural thickness was varied. However, the 0.4 mm sample lacked structural stability at the kirigami slit junction, resulting in uncontrolled buckling directions (red region). In contrast, the 2 mm sample exhibited no tilting deformation, preventing programmed origami activation (red region).

**
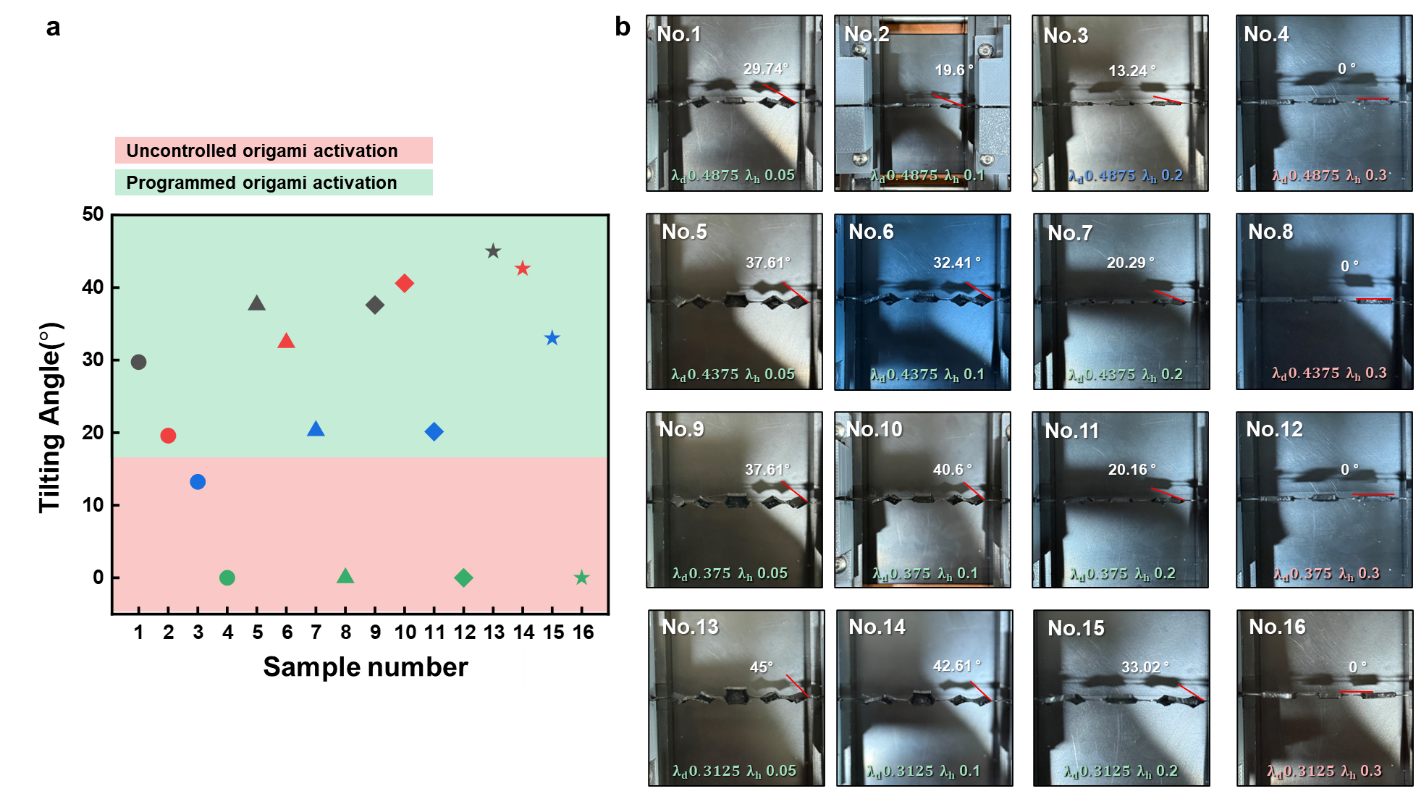
**

**Figure S5.** Design window for slit geometry enabling programmed origami activation:

(a) Graph showing the slit design range in which programmed origami activation occurs. The slit parameters were defined as $\lambda_{h}$= $h$/( $l_{u}$/2) and $\lambda_{d}$= $d$/$w_{u}$, as illustrated in Figure S3(b). For each condition, two samples were fabricated and tested for 50 repeated cycles. Green regions indicate conditions where the intended origami activation mode was consistently achieved throughout all cycles, whereas red regions denote conditions where the activation behavior was inconsistent.

(b) Tensile deformation behaviors observed for each slit configuration. Samples No.4, 8, 12, and 16 exhibited only in-plane deformation and failed to produce programmed origami activation (red region, 0%). Sample No.3 showed a tilting angle of approximately 13°, but the designed origami activation was not reliably achieved (red region).

These observations suggest that a sufficiently large tilting angle is associated with stable origami activation. Considering that the 1.8 mm thick sample in Figure S4 exhibited a tilting angle of approximately 17° with stable activation during repeated cycles, the experimental results suggest that slit geometries capable of inducing tilting angles above approximately 17° may be favorable for reliable programmed origami activation.

**
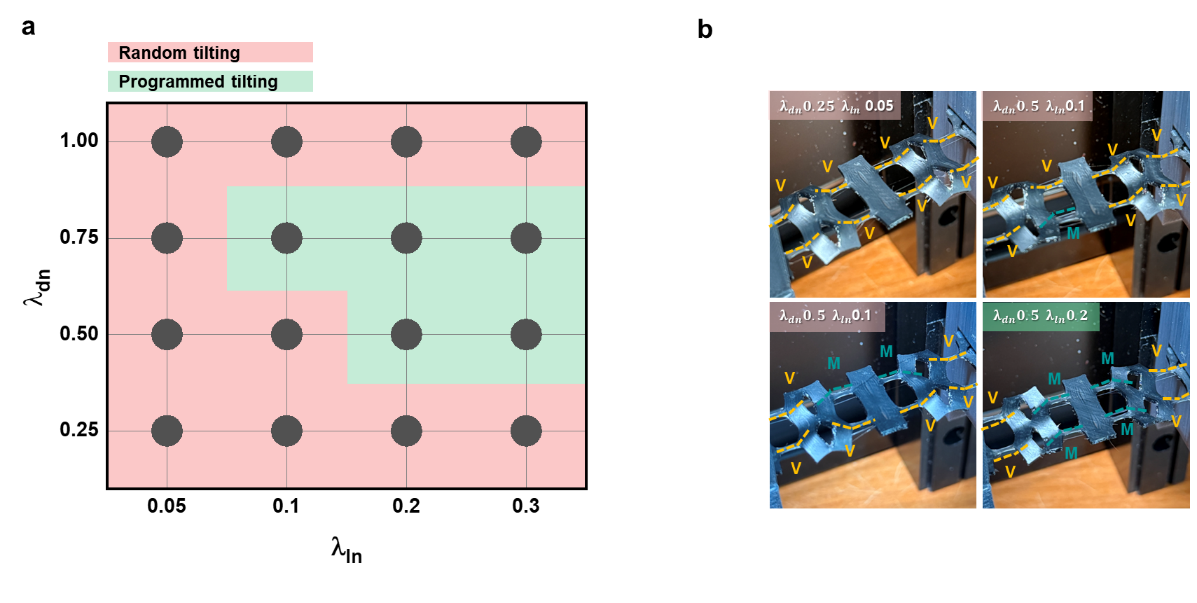
**

**Figure S6.** Design window for notch geometry enabling controlled deformation:

(a) Design window showing the conditions under which buckling can be directionally controlled by the notch geometry. The notch parameters were defined as $\lambda_{ln}$= $l_{n}$/( $l_{u}$/2) and $\lambda_{dn}$= $d_{n}$/$t_{u}$, as illustrated in Figure S3(b). For each condition, five samples were tested under tensile loading. A condition was classified as controlled deformation when all kirigami units consistently exhibited the programmed tilting direction across all tested samples (green region).

(b) Representative examples of successful and failed cases. In successful cases (green region), the tilting direction follows the designed valley (V) and mountain (M) configuration. In contrast, failed cases (red region) exhibit randomly oriented tilting, resulting in uncontrolled deformation.

These observations suggest a notch parameter range that enables directional tilting, which is associated with programmed origami activation.


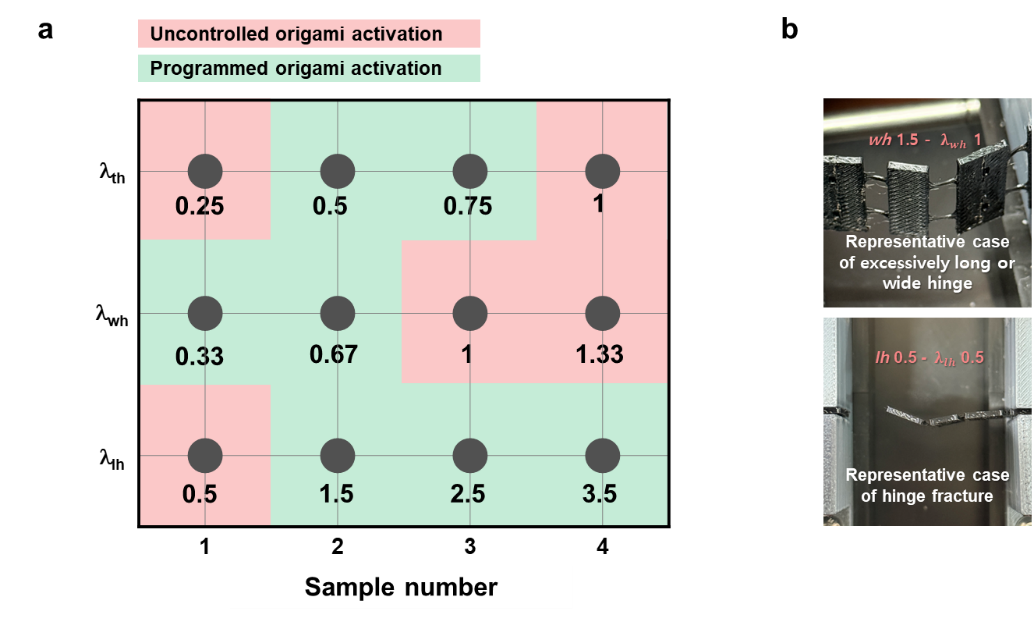


**Figure S7.** Design window for hinge geometry.

(a) Design window showing the conditions under which programmed origami activation occurs depending on hinge geometry. The hinge parameters were defined as $\lambda_{th}=t_{h}/t_{u}$*,* $\lambda_{wh}=w_{h}/l_{h}, \lambda_{lh}=l_{h}/2h$ as illustrated in Figure S3(b).

(b) Representative failure cases (red region). When the hinge length or width becomes excessively large, tensile loads cannot be effectively transferred, preventing the intended deformation. Conversely, when the hinge structure is mechanically weak, fracture occurs during tensile deformation.

These results suggest that the hinge geometry provides a relatively broad design window.


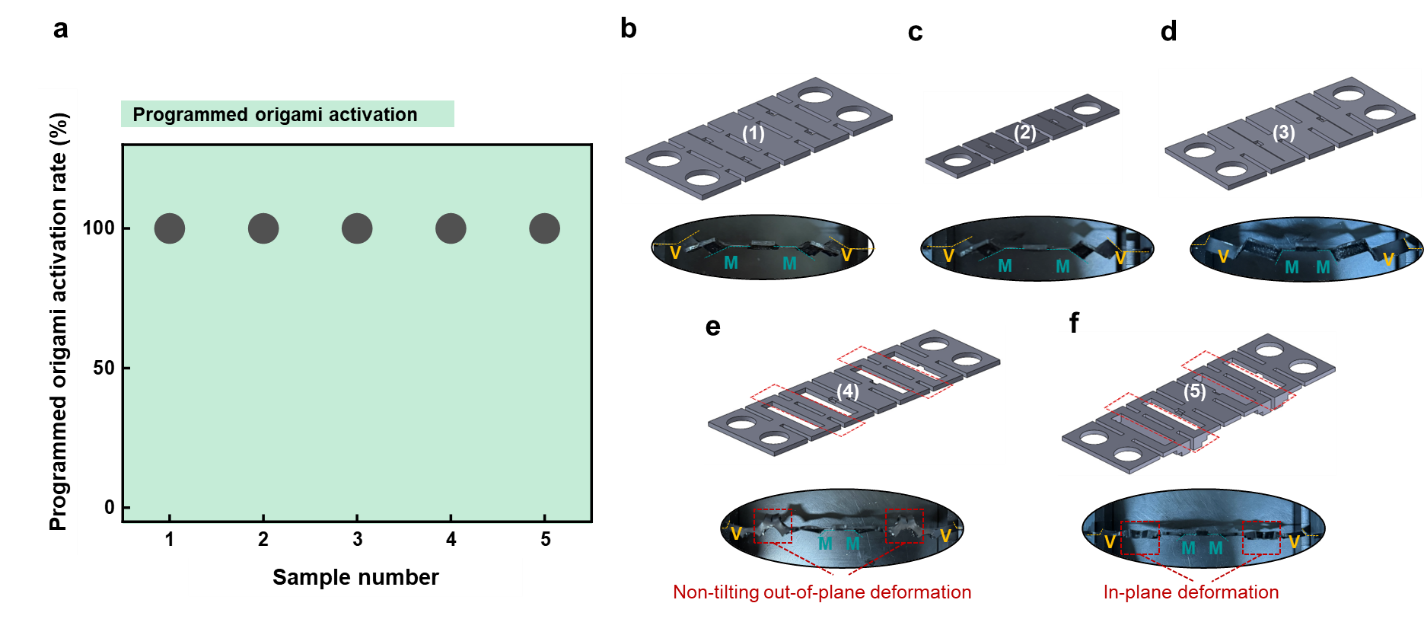


**Figure S8.** Application designs within the programmable origami design window.

(a) Design window illustrating representative application structures implemented within the experimentally identified parameter range (Figures S4–S7).

(b) Representative 323-slit kirigami structure and its tilting deformation under tensile loading.

($\lambda_{d}=$0.4875, $\lambda_{h}$ = 0.05, $\lambda_{th}=$0.5, $\lambda_{wh}=$0.67, $\lambda_{lh}=$1.5, $\lambda_{ln}=$0.2, $\lambda_{dn}=$0.5**)**

(c) Representative 212-slit kirigami structure and its tilting deformation under tensile loading.

($\lambda_{d}=$0.4875, $\lambda_{h}$ = 0.05, $\lambda_{th}=$0.5, $\lambda_{wh}=$0.67, $\lambda_{lh}=$1.5, $\lambda_{ln}=$0.4, $\lambda_{dn}=$0.5**)**

(d) Elongated 212-slit kirigami configuration and the corresponding tilting behavior during stretching. ($\lambda_{d}=$0.4875, $\lambda_{h}$ = 0.05, $\lambda_{th}=$0.5, $\lambda_{wh}=$0.67, $\lambda_{lh}=$1.5, $\lambda_{ln}=$0.2, $\lambda_{dn}=$0.5**)**

(e) Application design enabling enhanced stretchability based on the 212-slit kirigami architecture. ($\lambda_{d}=$0.3125, $\lambda_{h}$ = 0.2, $\lambda_{th}=$0.5, $\lambda_{wh}=$0.67, $\lambda_{lh}=$1.5, $\lambda_{ln}=$0.2, $\lambda_{dn}=$0.5**)**

(f) Stiffness-controlled application design derived from the 212-slit kirigami structure, demonstrating tunable deformation behavior.

($\lambda_{d}=$0.3125, $\lambda_{h}$ = 0.2, $\lambda_{th}=$0.5, $\lambda_{wh}=$0.67, $\lambda_{lh}=$1.5, $\lambda_{ln}=$0.2, $\lambda_{dn}=$0.5**)**

All structures were designed within the experimentally identified design window, suggesting that diverse kirigami configurations can exhibit programmed origami activation when the geometric parameters fall within the identified parameter range.

**
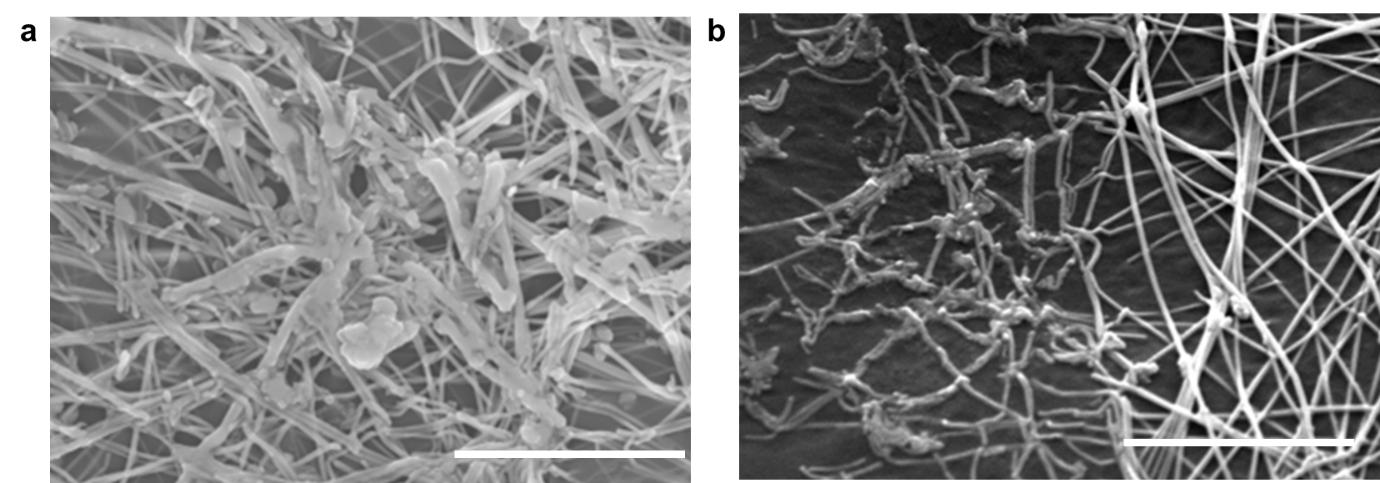
**

**Figure S9.** Surface morphology of AgNW networks after EGaIn application, with and without nano-root structures.

(a) With nano-root structures, the AgNW network remains uniformly distributed and maintains continuous conductive pathways after EGaIn application.

(b) Without nano-root structures, AgNW aggregation and local network disruption are observed after EGaIn application, resulting in discontinuous conductive pathways and reduced connectivity. Scale bars: 2 μm.

**
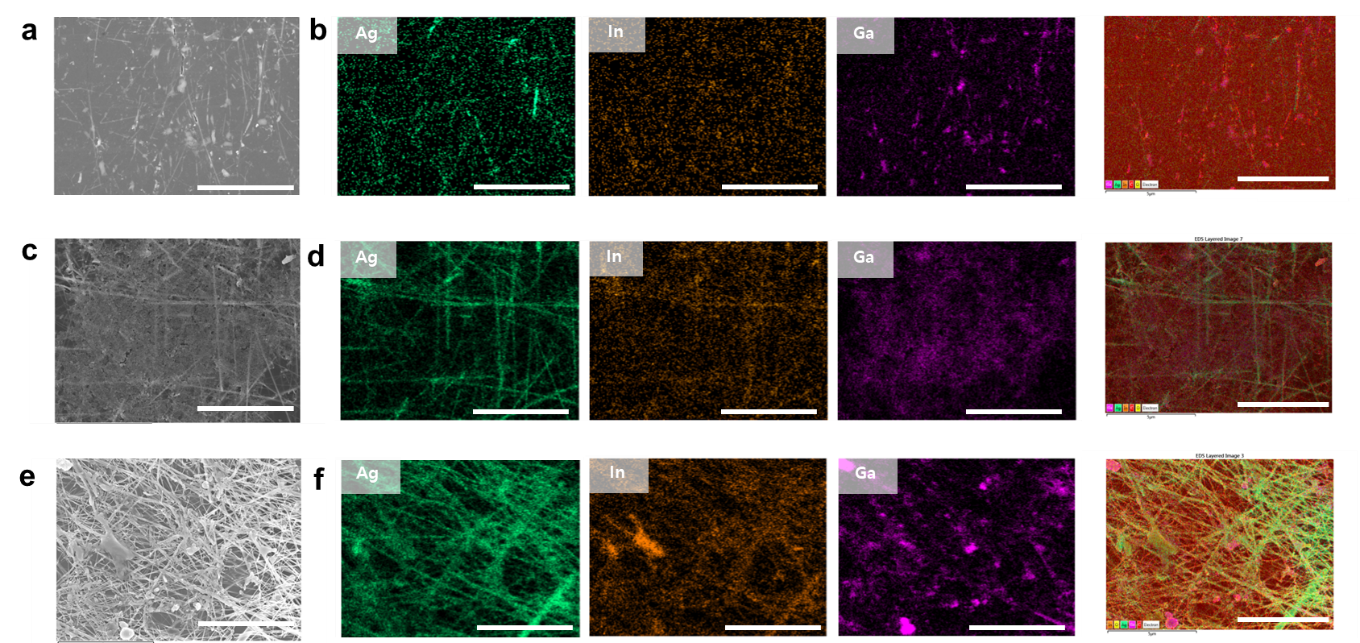
**

**Figure S10.** SEM images and EDS elemental mappings of AgNWs–In–Ga multiphasic alloy conductors prepared with different AgNW coating cycles.

(a,c,e) SEM images of the conductors prepared with (a) one, (c) two, and (e) four AgNW coating cycles.

(b,d,f) Corresponding EDS elemental maps (Ag, In, and Ga) for (b) one, (d) two, and (f) four coating cycles. Scale bars: 5 μm.


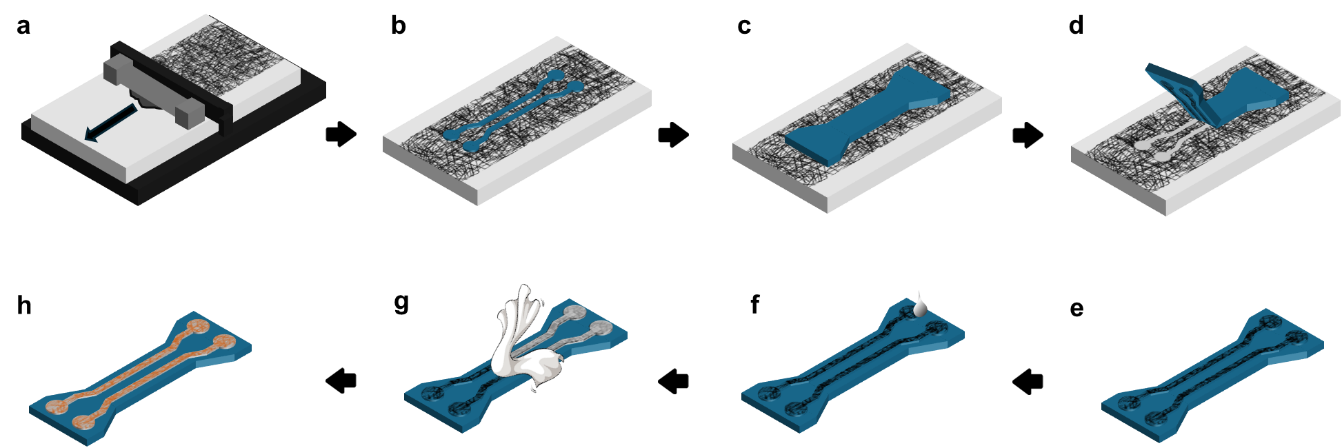


**Figure S11.** Fabrication process of the AgNWs–In–Ga multiphasic alloy conductor.

(a) AgNWs are coated onto glass substrates using a four-sided applicator mounted on an automatic bar coater.

(b,c) The AgNW-coated glass substrate is placed on the platform of an FDM 3D printer for direct printing of the transfer layer and the integration base.

(d,e) After printing, the AgNW-embedded structure is peeled off from the glass substrate, thereby transferring the AgNW network onto the printed base.

(f,g) EGaIn is deposited using a syringe and then uniformly spread by mechanical rupturing.

(h) The final AgNWs–In–Ga multiphasic alloy conductor is obtained after EGaIn application and localized AgNW–In interfacial alloying within the conductive network.


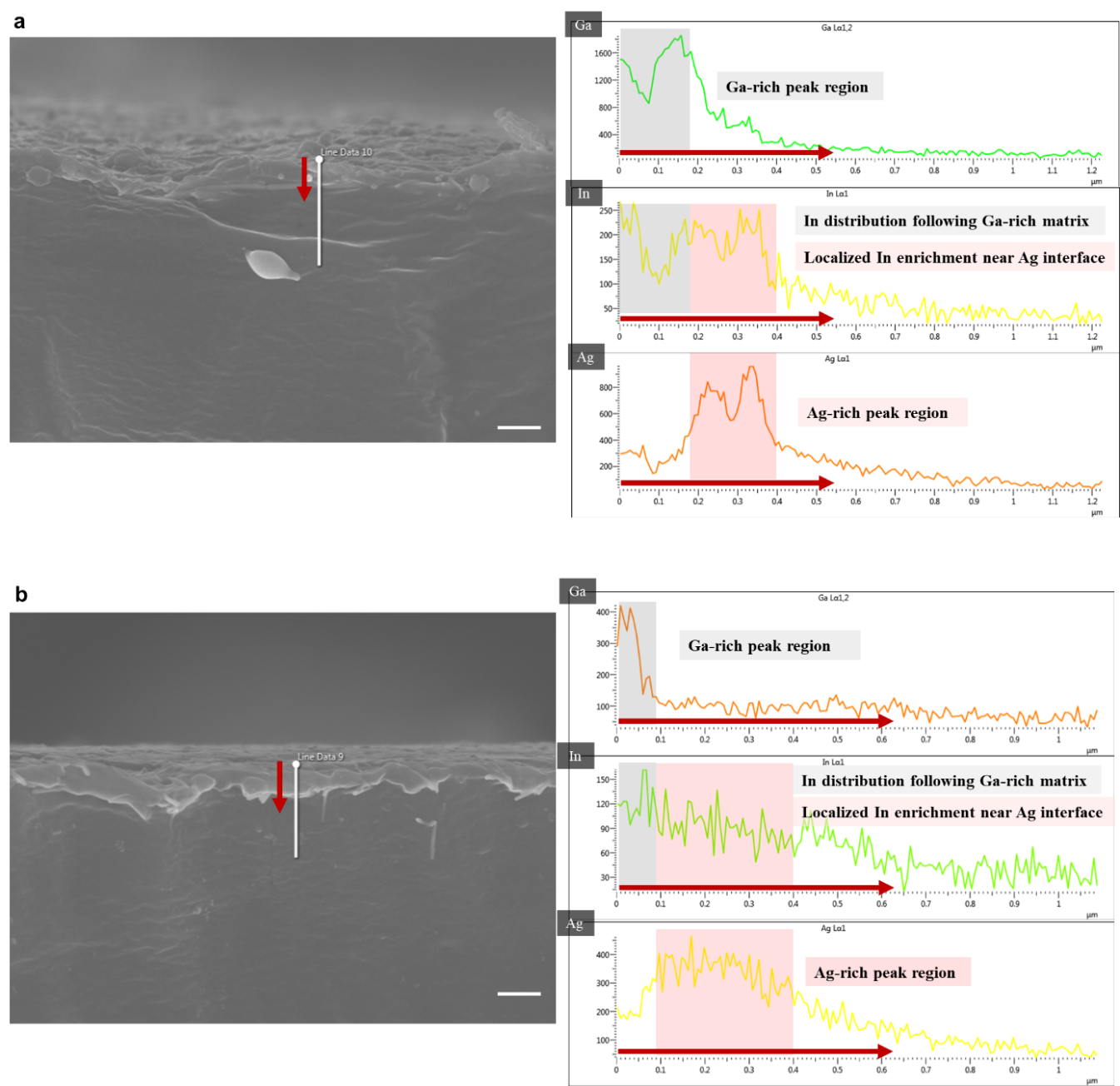


Figure S12. Cross-sectional EDS line-scan analysis showing correlated elemental distributions within the AgNWs–In-Ga multiphasic alloy.

(a) Line-scan profile obtained from AgNWs–In-Ga multiphasic alloy with double nanowire coating.

(b) Line-scan profile obtained from electrodes with single nanowire coating.

In both cases, indium signals exhibit partial correlation with both Ag-rich and Ga-rich regions. Indium enrichment appears near the Ag-rich peak region, suggesting localized AgNW–In interfacial interactions, while the broader distribution of indium following the Ga-rich region is consistent with the presence of a Ga–In liquid matrix. These observations are consistent with a multiphasic AgNWs–In–Ga multiphasic alloy conductive network involving localized interfacial alloying.

Red arrows indicate the line-scan direction and the analyzed distance. Scale bars: 500 nm.


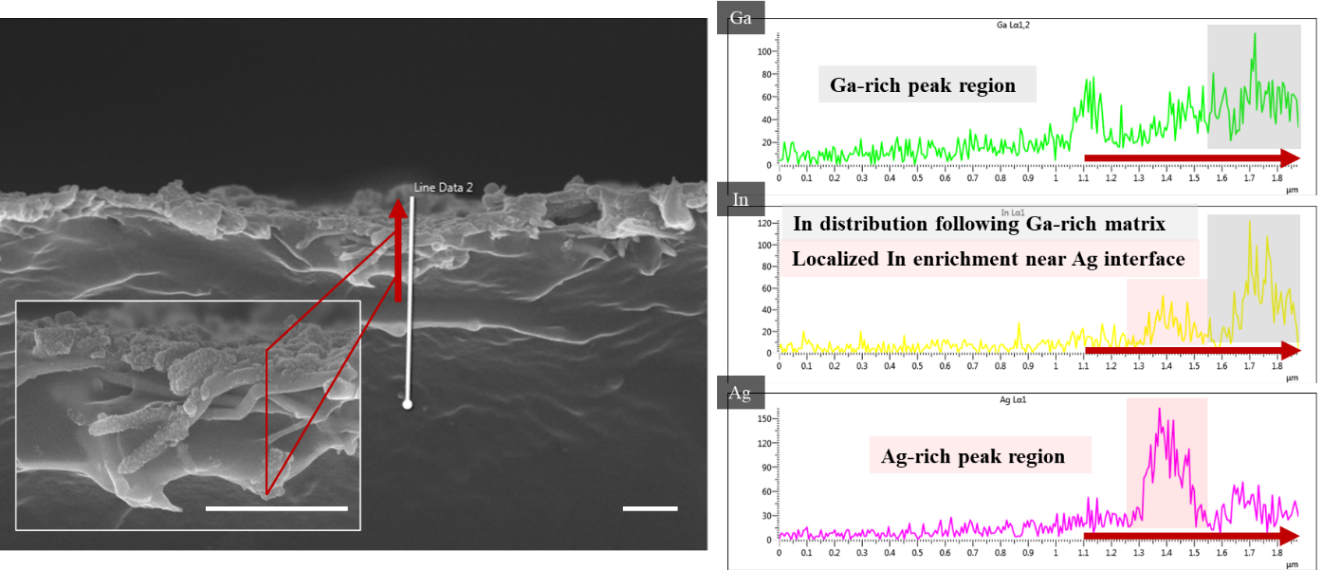


**Figure S13.** Cross-sectional EDS line-scan analysis of the AgNWs–In-Ga multiphasic alloy after acetone rinsing.

The residual structures remaining after removal of excess liquid metal exhibit correlated distributions of indium with both Ag-rich and Ga-rich regions, suggesting that indium remains associated with both the Ag interface and the surrounding Ga–In matrix. In addition, EDS line-scan analysis performed after acetone rinsing shows that the spatial distributions of Ga, In, and Ag retain concentration peak patterns similar to those observed prior to rinsing. These observations suggest that the elemental distributions are not solely attributable to residual liquid metal and are consistent with interfacial interactions at the AgNWs–liquid metal interface.

High-magnification SEM imaging further indicates that the nanowire mesh network structure remains largely preserved after rinsing. No obvious continuous reaction layer or uniform coating is observed along the nanowires. Instead, localized nanoscale particulate features appear near the interface regions, suggesting that the reaction products may be confined to discrete nanoscale domains rather than forming a continuous layer.

Red arrows denote the line-scan direction and analyzed distance. Scale bars: 500 nm.

**
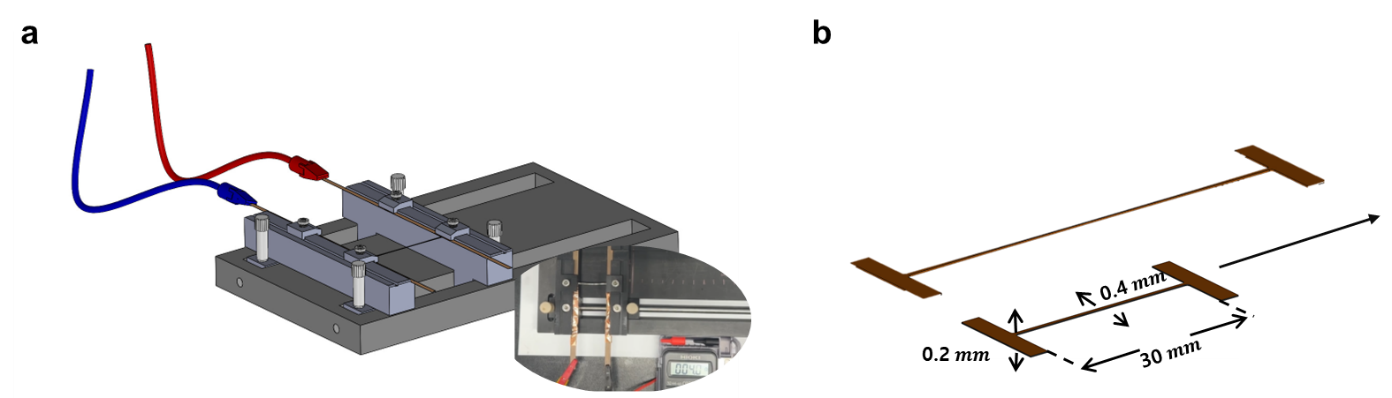
**

**Figure S14.** Schematic illustration of the tensile testing setup used to measure relative resistance change under tensile strain.

(a) Three-dimensional schematic of the tensile testing configuration corresponding to the actual measurement setup, together with a photograph of the actual test setup. The specimen was mounted between mechanical clamps connected to a displacement-controlled stage, enabling controlled stretching while maintaining electrical connection through direct ohmic contact. The sample was mounted on a fixing stage that simultaneously provides mechanical clamping and electrical connection through direct ohmic contact, ensuring a stable measurement environment.

(b) Schematic of the specimen used for stretching and folding tests.

**
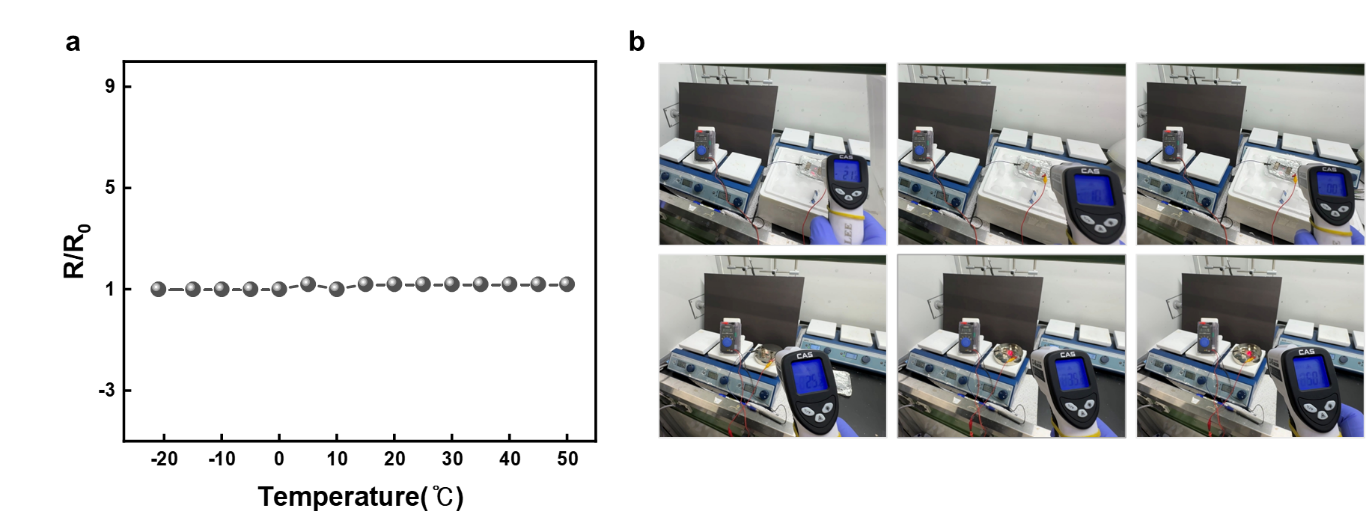
**

**Figure S15.** Measured electrical resistance of the nano-root anchored AgNWs–In–Ga multiphasic alloy conductor under varying temperature.

(a) Relative resistance change as a function of temperature.

(b) Photograph of the temperature measurement setup used for the experiment.

**
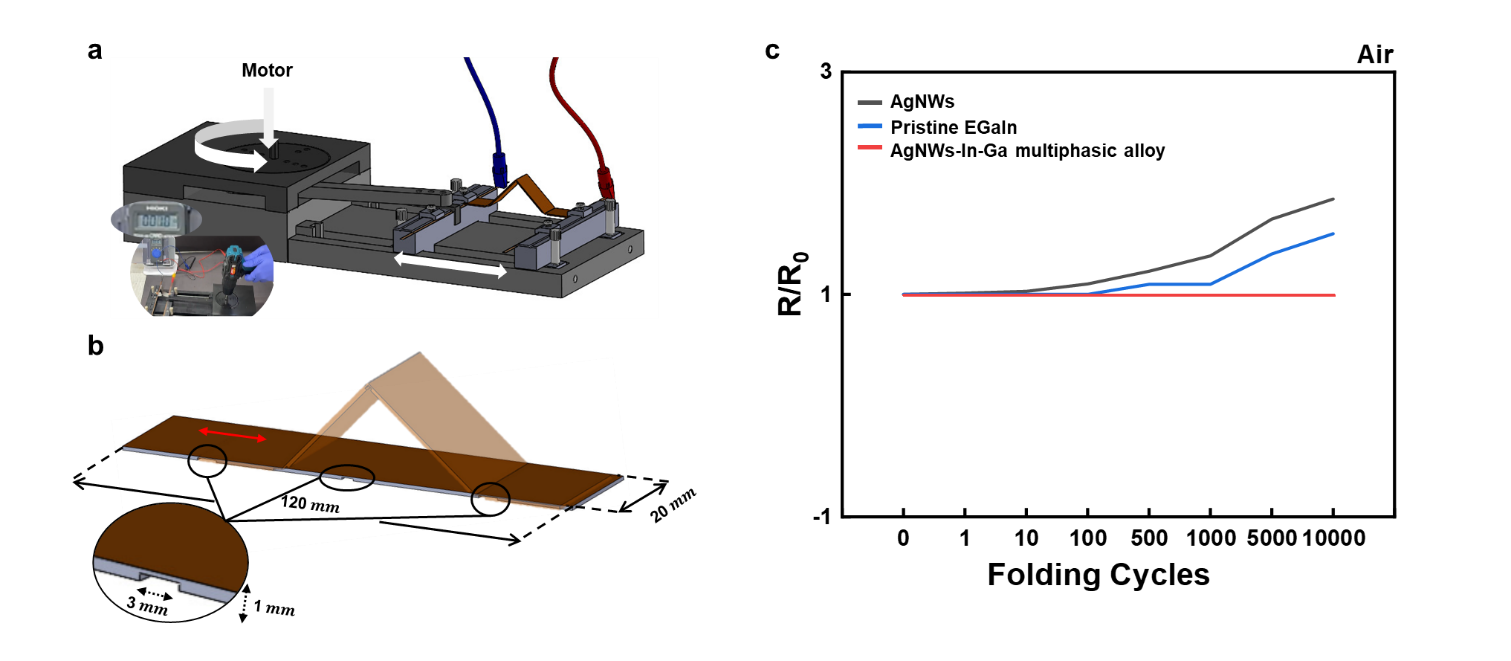
**

**Figure S16.** Cyclic folding test under ambient conditions.

(a) Three-dimensional schematic of the folding test setup corresponding to the actual experimental configuration, together with a photograph of the actual test setup (initial resistance ≈ 1.0). In this system, rotational motion generated by the motor is converted into a sliding displacement, enabling repeated folding of the specimen. The sample was mounted on a fixing stage that simultaneously provides mechanical clamping and electrical connection through direct ohmic contact, ensuring a stable measurement environment. The cyclic folding motion was performed at a sliding speed of 450 mm s⁻¹.

(b) Schematic illustration of the electrode used in the folding test.

(c) Relative resistance change (ΔR/R₀) during cyclic folding under ambient conditions for AgNWs (gray), pristine EGaIn (blue), and the nano-root–anchored AgNWs–In–Ga multiphasic alloy conductor (red).

**
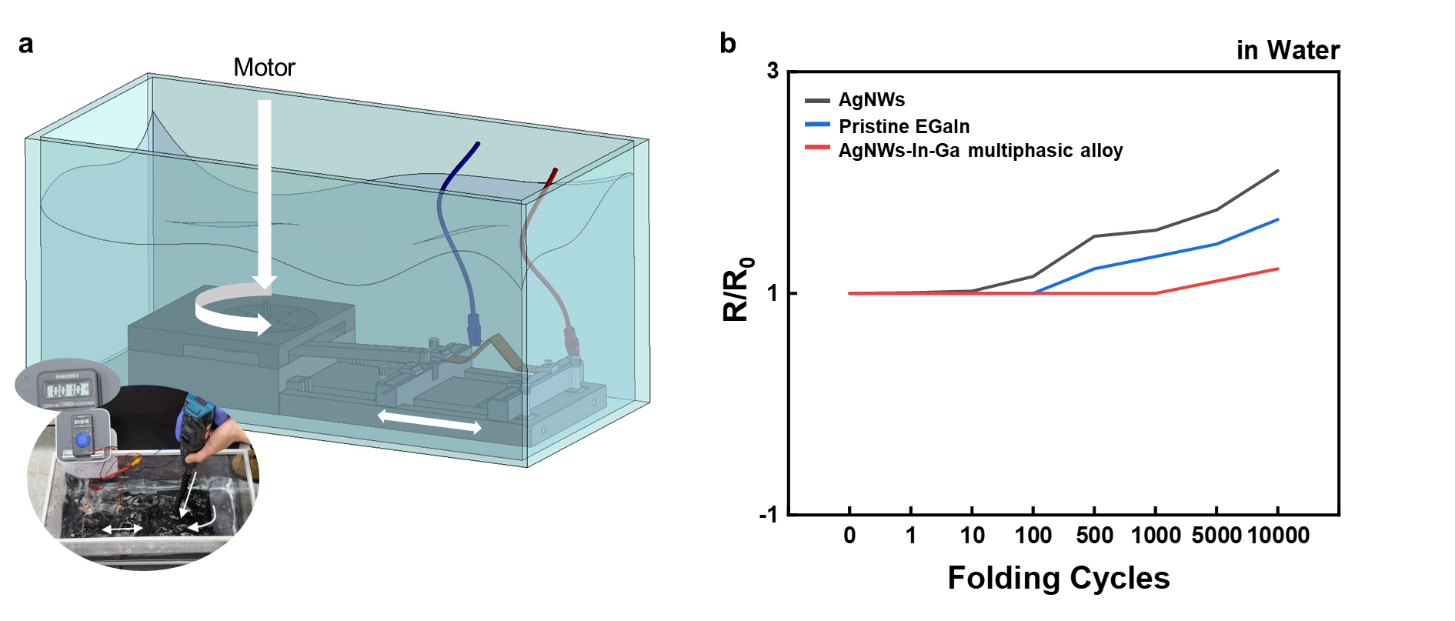
**

**Figure S17.** Cyclic folding test under underwater conditions .

(a) Three-dimensional schematic of the folding test setup corresponding to the actual experimental configuration, together with a photograph of the actual test setup under underwater conditions (initial resistance ≈ 1.0). In this system, rotational motion generated by the motor is converted into a sliding displacement, enabling repeated folding of the specimen. The sample was mounted on a fixing stage that simultaneously provides mechanical clamping and electrical connection through direct ohmic contact, ensuring a stable measurement environment. The cyclic folding motion was performed at a sliding speed of 450 mm s⁻¹.

(b) Schematic illustration of the electrode used in the folding test.

(c) Relative resistance change (ΔR/R₀) during cyclic folding under underwater conditions for AgNWs (gray), pristine EGaIn (blue), and the nano-root–anchored AgNWs–In–Ga multiphasic alloy conductor (red).

**
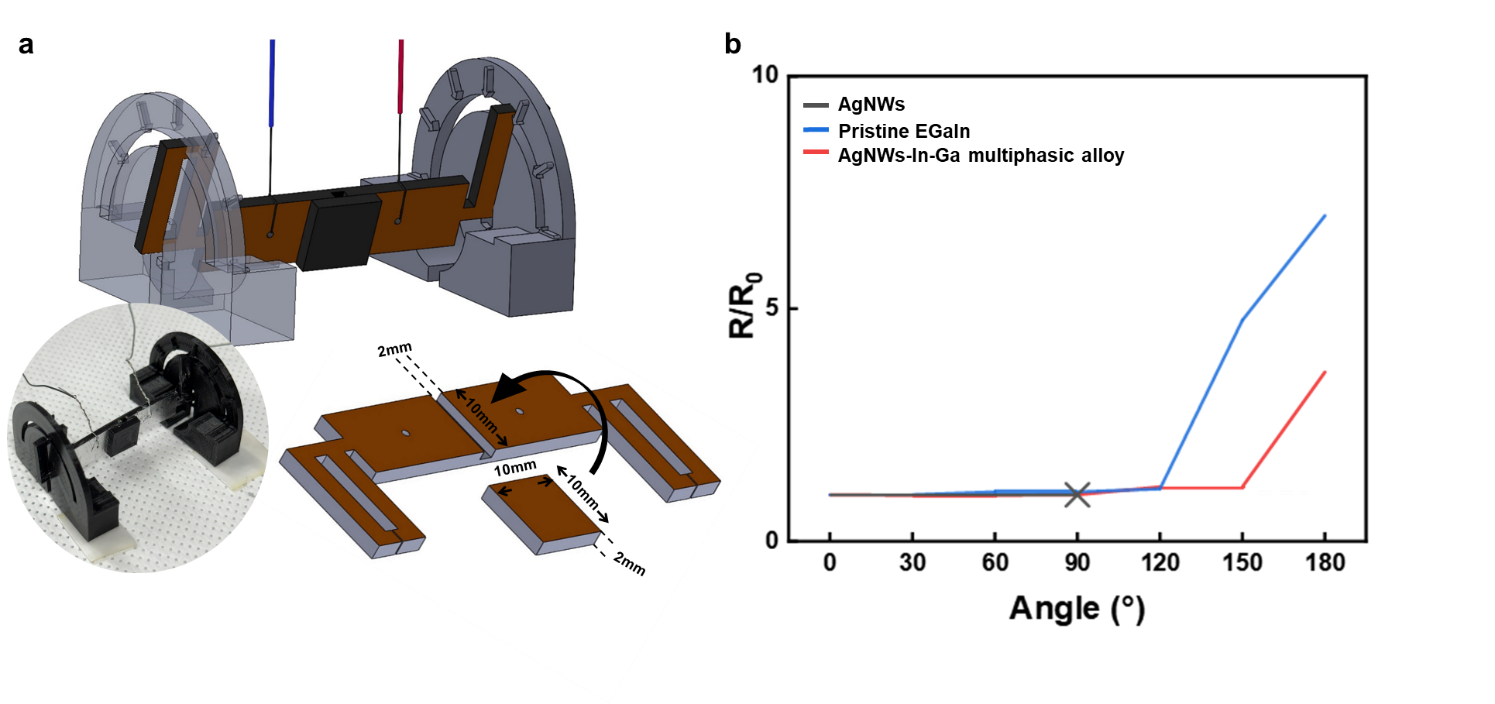
**

**Figure S18.** Rotation test and corresponding resistance variation as a function of rotation angle.

a)Three-dimensional schematic of the rotation test setup corresponding to the actual experimental configuration, together with a photograph of the actual test setup. Rod-shaped electrodes with an electrically disconnected center region were fabricated. Electrical continuity between the two ends was restored by attaching a 205.9 mg block carrying an identical electrode pattern. The block was rotated in 30° increments, and the electrical resistance between the two ends was measured using a multimeter connected through external wiring.

b) Relative resistance change (ΔR/R₀) during rotation test for AgNWs (gray), pristine EGaIn (blue), and the nano-root–anchored AgNWs–In–Ga multiphasic alloy conductor (red).


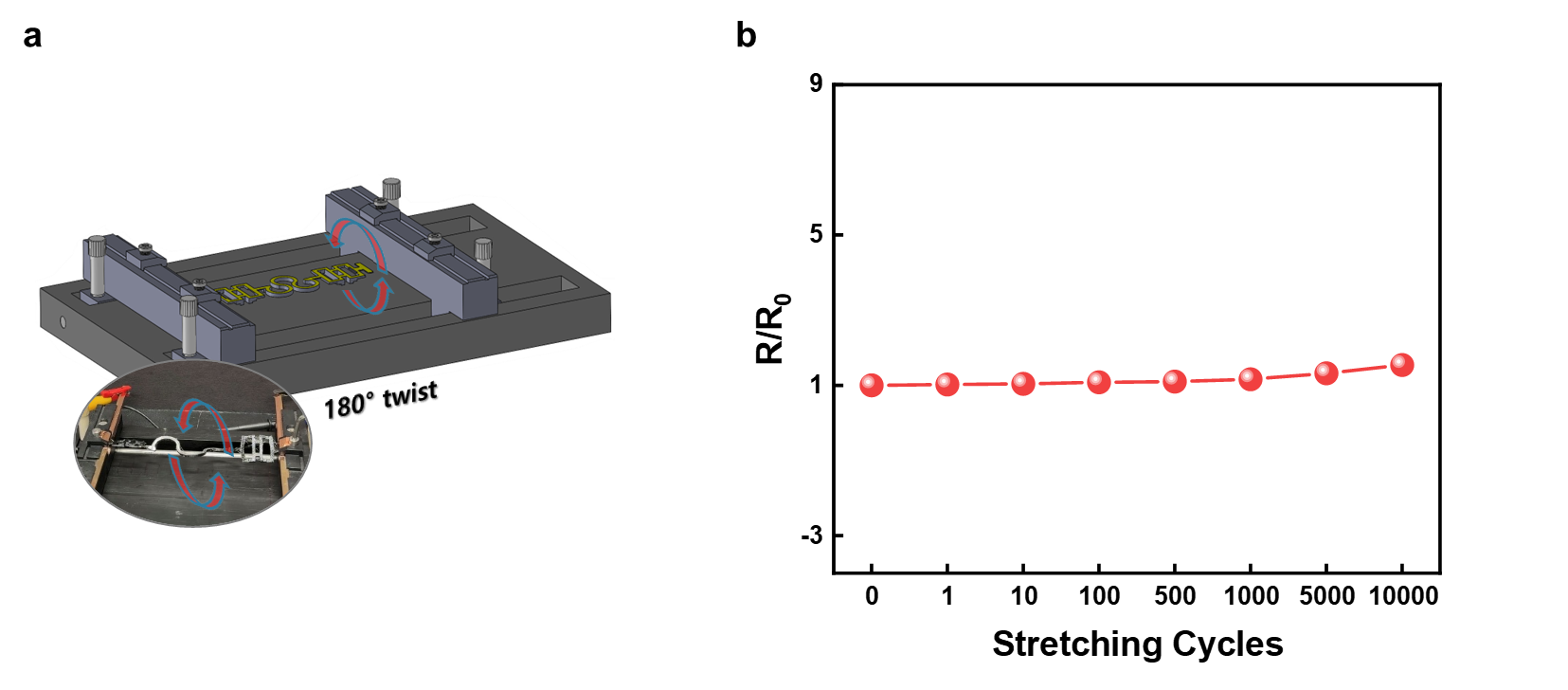


**Figure S19.** Electrical stability of the electrode under cyclic tensile loading in a twisted configuration.
(a) Three-dimensional schematic of the cyclic tensile loading test setup corresponding to the actual experimental configuration and the corresponding photograph of the twisted sample mounted on the measurement stage. A serpentine-based structure capable of accommodating twisted configurations was employed, and cyclic tensile loading up to 60% strain was applied, corresponding to the maximum tensile strain range of the serpentine geometry. The sample was mounted on a fixing stage that simultaneously provides mechanical clamping and electrical connection through direct ohmic contact, ensuring a stable measurement environment.
(b) Relative resistance change (ΔR/R₀) measured during 10,000 tensile cycles.

These results demonstrate that the electrode maintains stable electrical performance even under repeated tensile deformation in a twisted configuration.


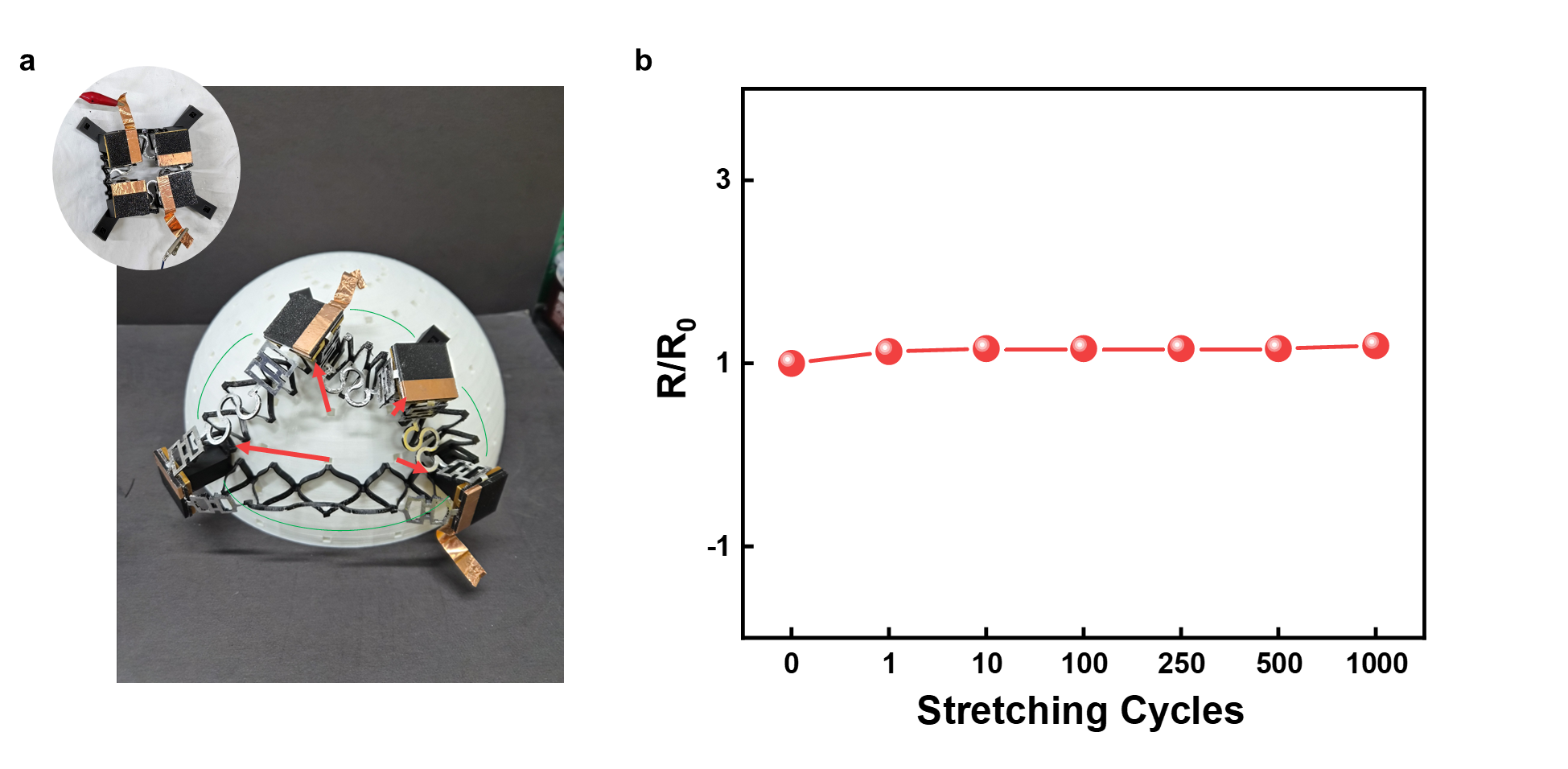


**Figure S20.** Electrical stability under repeated deformation under curvature and off-axis loading conditions.
(a) Experimental configuration used to evaluate electrical stability under multiaxial deformation conditions. The system was mounted on a hemispherical substrate with regularly arranged fixation slots, enabling deformation under both curvature and off-axis loading conditions. The electrical connection used for resistance measurements is also shown.
(b) Relative resistance change (ΔR/R₀) measured during 1000 deformation cycles.

These results demonstrate that the system maintains stable electrical performance even under repeated deformation involving both curvature and off-axis loading.


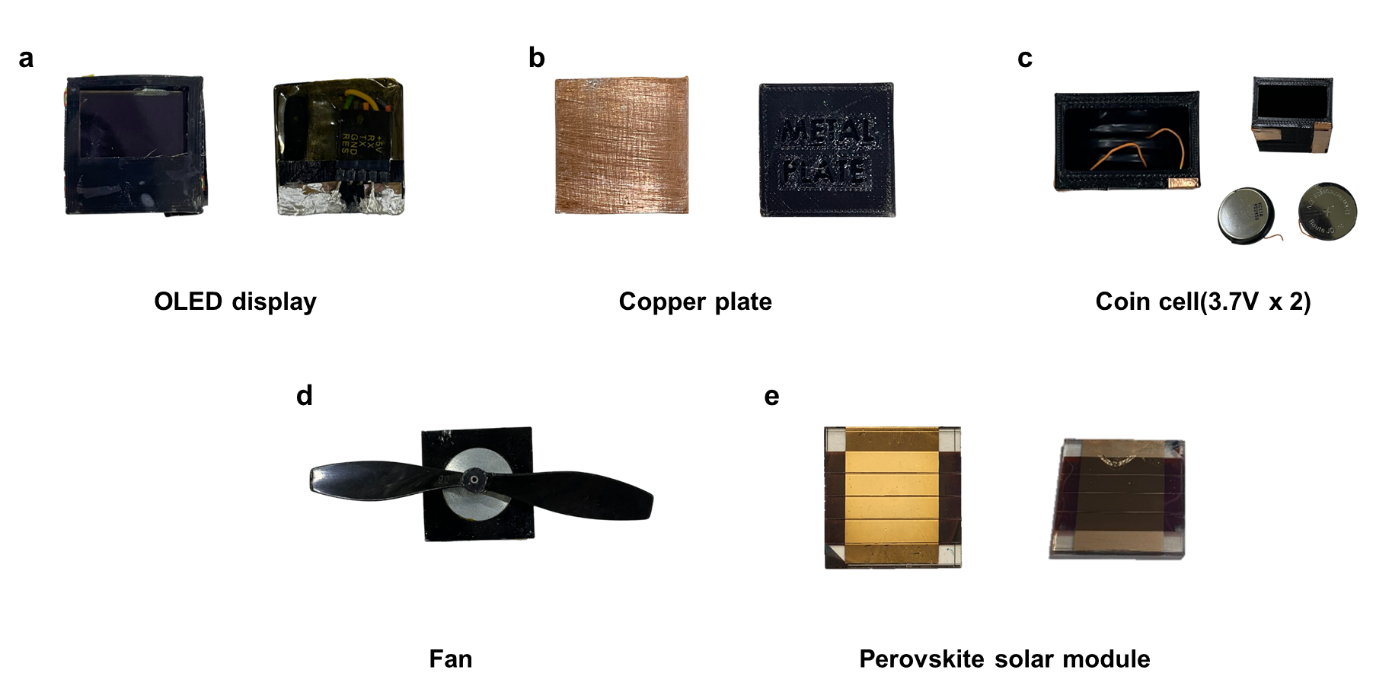


**Figure S21.** Devices used in the reversible stack-and-connect demonstration.

(a) Front and back views of the OLED display.

(b) Front and back views of the substrate with the attached copper plate.

(c) Front and back views of the battery module used in the demonstration.

(d) Fan.

(e) Front view of the perovskite PV module.


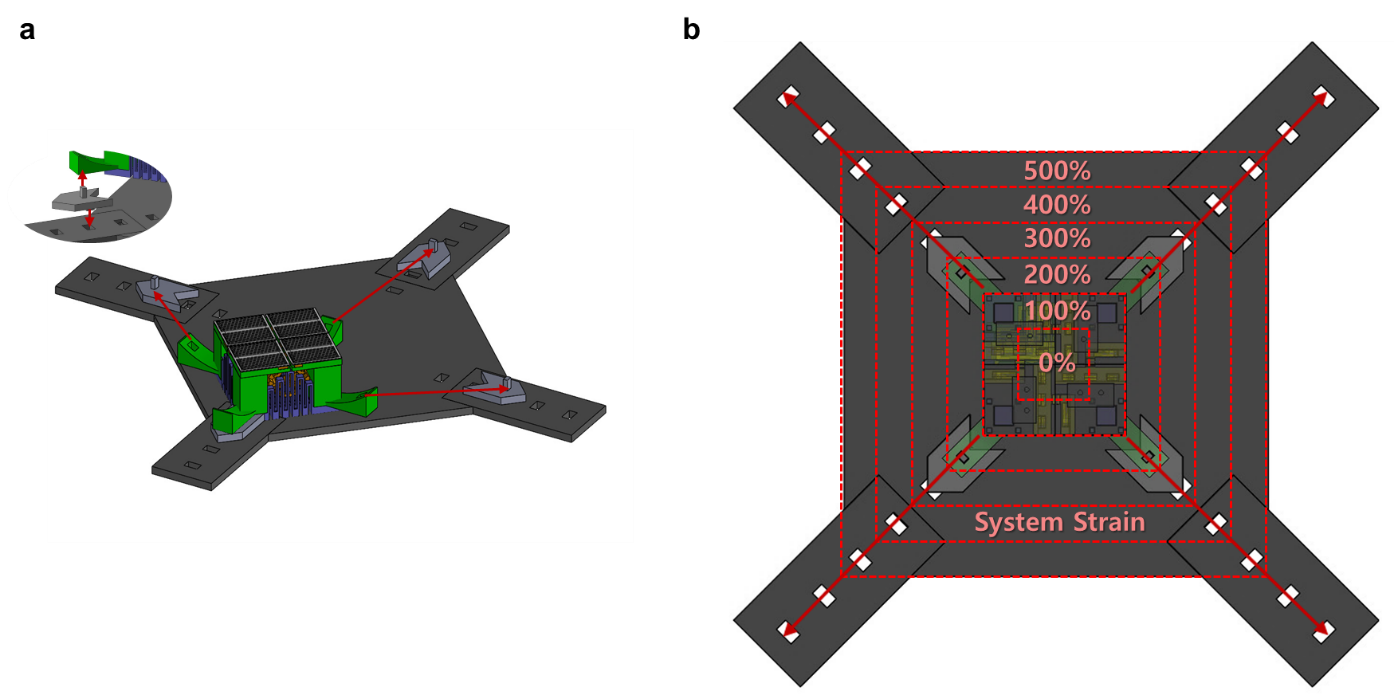


**Figure S22.** Experimental setup and definition of system strain for evaluating 500% stretchability.
(a) Three-dimensional schematic corresponding to the actual experimental setup used for mechanical and electrical characterization of the stretchable photovoltaic system.
(b) Definition of system strain during stretching. The system strain (ε_sys) was defined based on the change in the center-to-center distance between adjacent photovoltaic modules (rigid islands) along the stretching direction, measured between the geometric centers of the photovoltaic devices. Specifically,

$$\varepsilon_{sys}=\frac{L-L_{0}}{L_{0}}\times100\%$$

where $L_{0}$and $L$represent the initial and stretched center-to-center distances between neighboring photovoltaic devices, respectively, extracted from top-view images. The centroid positions of individual modules were determined through image-based geometric analysis. The dashed squares indicate representative system strain levels ranging from 0% to 500%.

**
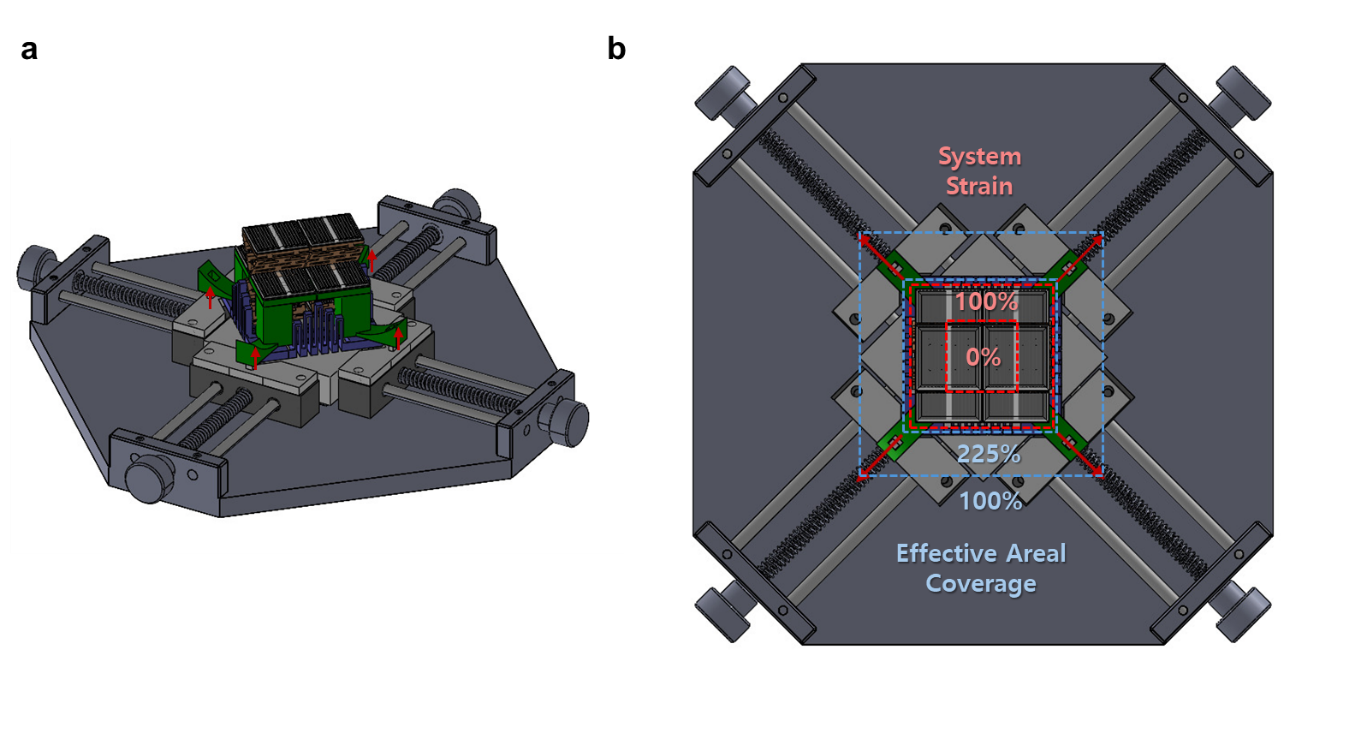
**

**Figure S23.** Experimental setup and definition of effective areal coverage.

(a) Three-dimensional schematic corresponding to the actual experimental setup used for evaluating system strain and effective areal coverage of the stretchable photovoltaic system.
(b) Definition of effective areal coverage during deformation. The areal coverage ($C_{A}$) was defined as the ratio between the total area of photovoltaic devices present within the system and the projected footprint area of the array:

$$C_{A}=\frac{A_{tot}}{A_{fp}}\times100\%$$

where $A_{tot}$represents the total area of all photovoltaic devices integrated within the system, and $A_{fp}$denotes the footprint area of the array. Because the photovoltaic modules share a three-dimensional spatial envelope within the structure, part of the device area is initially accommodated in a hidden configuration, resulting in an initial effective areal coverage of approximately **225%** relative to the footprint area. Based on this definition, as illustrated in Figure 6g, the projected footprint of the system can expand to 100% system strain while maintaining over 100% effective areal coverage. These definitions enable consistent quantification of the stretchability–areal coverage decoupling enabled by the three-dimensional kirigami architecture.


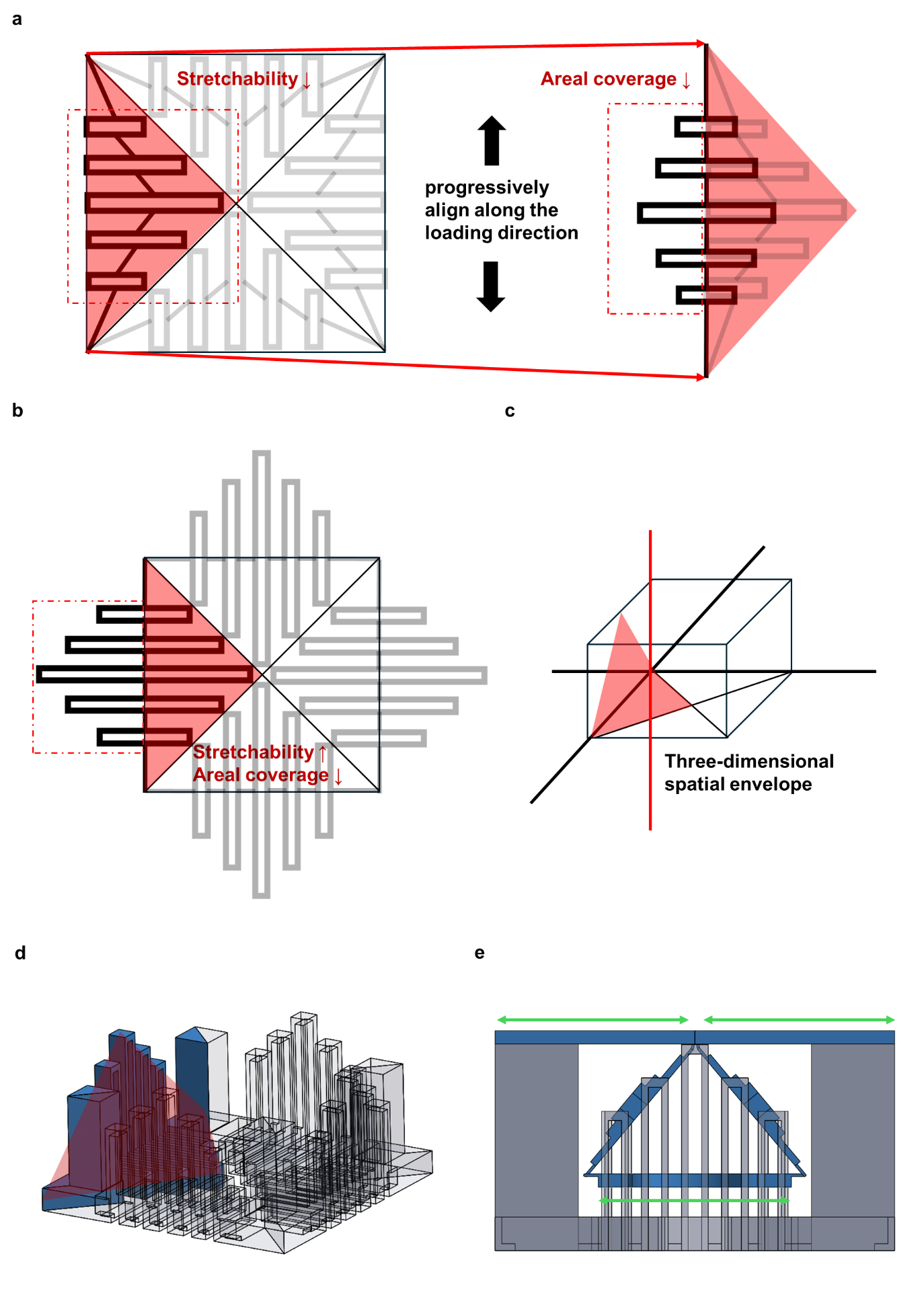


**Figure S24.** Design strategy of the substructure and associated geometric constraints.

(a) Conceptual illustration of a symmetric kirigami geometry introduced within a confined planar area. As tensile deformation progresses, the kirigami units progressively align along the loading direction, resulting in a reduction in effective areal coverage.

(b) Two-dimensional layout of the symmetric kirigami configuration when stretchability is maximized. Due to geometric packing characteristics, the area occupied by kirigami units increases, thereby reducing the achievable planar areal coverage.

(c) Conceptual representation of the three-dimensional spatial envelope required to accommodate symmetric kirigami structures within a limited planar footprint.

(d) 3D kirigami structure employing a three-dimensional symmetric kirigami configuration that expands spatial utilization beyond planar geometric constraints.

(e) Conceptual illustration shows that, when the rigid segments of the upper structure are designed with a 1:1:1 geometric ratio, hidden structural accommodation becomes possible within the confined spatial envelope.

To achieve both stretchability and structural recoverability, kirigami structures are typically required to adopt symmetric geometries. However, when symmetric kirigami patterns are introduced within a confined planar footprint that must be shared among asymmetric functional components, an inherent trade-off arises between stretchability and areal coverage.

To overcome this limitation, a three-dimensional kirigami architecture was designed to extend structural utilization beyond the geometric constraints of the two-dimensional footprint by exploiting the third spatial dimension. This 3D kirigami configuration enables large stretchability even within a limited planar area.

Furthermore, to accommodate identical energy devices within this confined spatial envelope, the upper structure was designed with a 1:1:1 geometric ratio, together with an arrangement strategy that enables efficient structural integration within the restricted footprint. Nevertheless, the accessible three-dimensional spatial volume remains fundamentally governed by the footprint area, which consequently acts as an inherent limitation when designing stretchability and areal coverage.


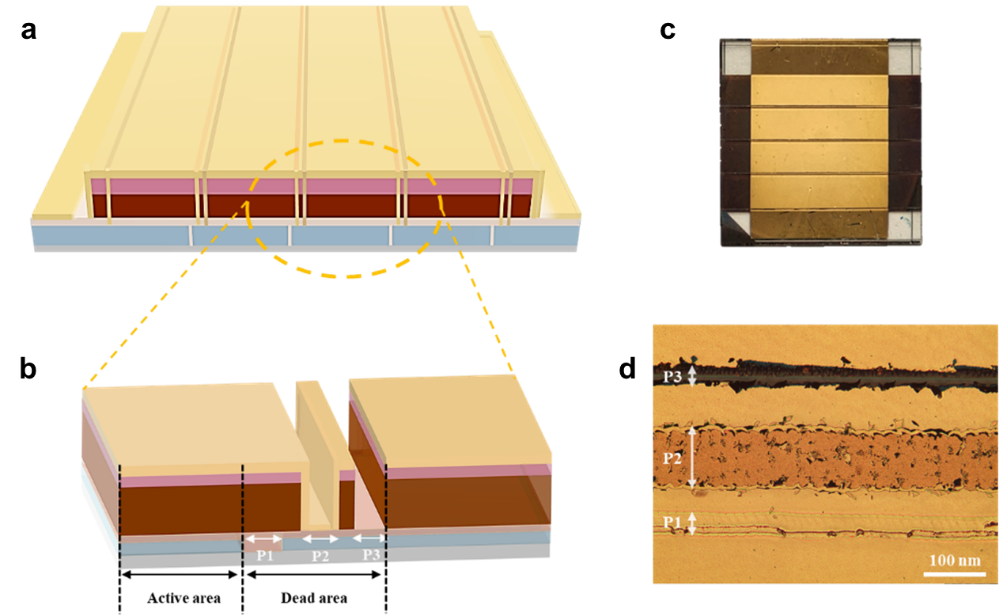


**Figure S25.** Structure and photographs of the fabricated perovskite solar module.

(a) Schematic illustration of the module interconnection layout.

(b) Enlarged schematic showing the P1, P2, and P3 scribing regions.

(c) Photograph of the completed 3 × 3 cm² perovskite solar module consisting of four series-connected cells.

(d) Optical micrograph of the scribed lines (P1, P2, and P3), highlighting the isolation regions for series interconnection.
